# RHBDF2 governs microglial neuroinflammation during cerebral ischemia–reperfusion injury and is positively regulated by the m6A reader YTHDF1

**DOI:** 10.1186/s10020-025-01326-y

**Published:** 2025-09-02

**Authors:** Lisi Xu, Ruonan Zhang, Xiaolin Zhang, Bing Liu, Xiuli Shang, Daifa Huang

**Affiliations:** 1 Department of the Second Cadre Ward, General Hospital of Northern Theater Command, Shenyang, China; 2 Department of Neurology, The First Hospital of China Medical University, Shenyang, China

**Keywords:** RHBDF2, Cerebral ischemia–reperfusion injury, Microglia, STING, RNA m6A methylation

## Abstract

**Background:**

Neuroinflammation mediated by microglia activation is the key pathological mechanisms for cerebral ischemia–reperfusion injury (CIRI). This study investigated the role and underlying molecular mechanism of Rhomboid 5 homolog 2 (RHBDF2) in neuroinflammation during CIRI.

**Methods:**

The in vivo middle cerebral artery occlusion and reperfusion (MCAO/R) mouse model and in vitro HMC3 microglia subjected to oxygen glucose deprivation and reperfusion (OGD/R) were established to mimic CIRI. Real-time PCR, western blot, immunohistochemistry, immunofluorescence, flow cytometry, and co-immunoprecipitation assays were used to confirm RHBDF2 expression and explore the molecular mechanism of microglia-specific RHBDF2 knockdown in CIRI. Methylated RNA immunoprecipitation was used to detect the m6A methylation level of RHBDF2 mRNA both in vivo and in vitro. RNA sequencing analysis was performed in OGD/R-treated HMC3 cells with or without RHBDF2 knockdown.

**Results:**

Our finding showed that RHBDF2 expression increased in both in vivo and in vitro CIRI models. Microglial-specific RHBDF2 knockdown reduced brain injury in MCAO/R mice, as evidenced by the reduction in the cerebral infarct volume and amelioration of the neurological deficits. Furthermore, we demonstrated that RHBDF2 knockdown alleviated neuroinflammation by inhibiting microglial M1 polarization and promoting microglial M2 polarization in MCAO/R mouse ischemic penumbra. Mechanistically, RHBDF2 interacted with STING and promoted the activation of the STING-TBK1-IRF3/p65 signaling pathway. Rescue experiments confirmed that RHBDF2 knockdown suppressed inflammation via the inhibition of STING-TBK1 signaling pathway. In addition, the m6A methylation level of RHBDF2 mRNA was significantly increased in the MCAO/R mouse brain tissues and OGD/R-treated HMC3 cells. YTHDF1 recognized the m6A sites of RHBDF2 and promote its expression in an m6A manner. Through RNA-seq, the possible downstream effectors of RHBDF2 in CIRI was predicted.

**Conclusions:**

Microglial-specific RHBDF2 knockdown inhibits neuroinflammation in CIRI via STING-TBK1 signaling pathway, and is positively regulated by the m6A reader YTHDF1. This suggests RHBDF2 as a potential therapeutic target in ischemic stroke.

**Graphical abstract:**

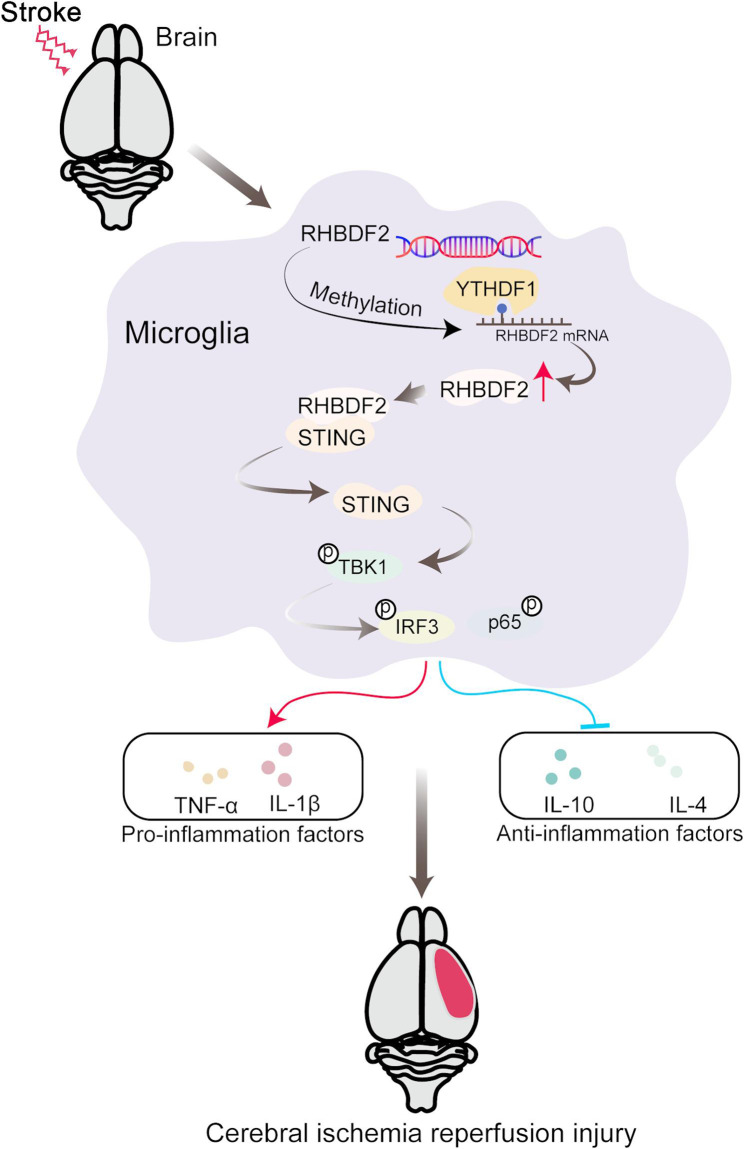

**Supplementary Information:**

The online version contains supplementary material available at 10.1186/s10020-025-01326-y.

## Background

Cerebral ischemic stroke, which is primarily caused by reduced blood flow to the brain resulting in brain tissue damage, is a leading cause of death and disability (Mendelson and Prabhakaran 2021). It is important to note that once blood flow is restored, a condition known as cerebral ischemia–reperfusion injury (CIRI) triggers a rapid sequence of neuropathological events (Chamorro et al. 2016). Recent reports have demonstrated that the cytokine-dependent microenvironment plays a critical role in the progression of CIRI (Jurcau and Simion 2021). Accumulating evidence suggested that innate immune and inflammatory responses, in particular microglial infiltration, contribute to the CIRI (Ma et al. 2017; Choi and Pile-Spellman 2018; Zhang et al. 2022). Microglia would be phenotypically polarized into two major phenotypes, M1 and M2, and are activated and concentrated in the peri-infarct area (Hu et al. 2012). M1 microglia release inflammatory mediators such as inducible nitric oxide synthase (iNOS) and interleukin (IL)-1β, which exacerbated brain damage (Shen et al. 2022). In contrast, M2 cells are characterized by secreting anti-inflammatory mediators including IL-4 and IL-10 that promote tissue repair (Li et al. 2023a). The inflammatory response in the brain after ischemia and reperfusion depends on the imbalance of M1/M2 cells and their ratio (Liu et al. 2021). Previous studies have shown that in the early stage of CIRI, the pro-inflammatory M1 phenotype is dominant in the peri-infarct region (Hu et al. 2012). Moreover, overactivated microglia activate various inflammatory pathways, such as nuclear factor-κB (NF-κB) pathway, which trigger the release of a variety of proinflammatory mediators, exacerbating acute inflammatory responses (Li et al. 2023b). Therefore, the exploration of novel immune regulation that balances microglial phenotypes in the acute ischemic penumbra may be a feasible strategy for the prevention or reduction of CIRI.

Rhomboid protein 2 (iRhom2), also known as RHBDF2, is an inactive member of the rhomboid family of serine proteolytic enzymes that plays a critical role in regulating protein degradation and inflammatory responses (McIlwain et al. 2012). Previous studies have suggested that RHBDF2 is a critical inflammation-associated mediator in the control of the release of proinflammation cytokine (Issuree et al. 2013). In lung ischemia and reperfusion injury, inhibition of RHBDF2 plays a protective role via inhibiting inflammation response (Kim et al. 2018a; Kim et al. 2020). In addition, the change of RHBDF2 expression in macrophage influenced the inflammation in cardiomyocyte under the metabolic stress (Ge et al. 2019). RHBDF2 has been verified to positively regulate the expression of stimulator of interferon gene (STING), which affected microglial polarization by affecting downstream IRF3/NF-κB expression (Lin et al. 2021; Zhao et al. 2023). Recent studies have suggested that RHBDF2 was expressed in the brain and localized to microglia (Li et al. 2020). In the late-onset Alzheimer's, RHBDF2 has been demonstrated as a modifier of microglial TREM2 proteolysis (Jocher, et al. 2025). An analysis of online databases revealed that RHBDF2 is predominantly expressed in the microglia of brain tissue. Inhibition of RHBDF2 effectively alleviated nerve damage by suppressing PM 2.5-induced inflammatory response (Xu et al. 2020). However, whether RHBDF2 functions in CIRI, in microglia-induced neuroinflammation, remains to be investigated.

N6-methyladenosine (m6A) modification functioned in the progression of CIRI has been reported (Yi et al. 2021). In the study of Jager et al*.,* they verified that there might have association between the methylation and RHBDF2 expression in nervous system (Jager et al. 2014). Given the emerging role of m6A modification in CIRI, its potential regulation of RHBDF2 remains unexplored.

In this study, we found that RHBDF2 expression was upregulated in CIRI. Microglial-specific RHBDF2 knockdown alleviated brain injury in CIRI, and further elucidated the underlying mechanism. Taken together, our findings suggest that RHBDF2 may function as a novel target in CIRI.

## Methods

### Data acquisition

Two microarray datasets (GSE61616 and GSE58720) detailing the gene expression profiles of CIRI and the microarray datasets (GSE106931) detailing the gene expression profiles of macrophage after CIRI were obtained from the Gene Expression Omnibus (GEO) database. The GSE61616 dataset contained five control and five ischemia samples. The GSE61616 samples were from the right hemispheres of male Sprague–Dawley rats that were subjected to 2 h of occlusion followed by 7 days of reperfusion. GSE106931 included sorted CD11b^+^CD163^+^ cells from rat brain tissue (from three control rats and three ischemic rats 16 h post-ischemia). GSE58720 contained mouse brain tissue samples from five control and five ischemia samples. The ischemia samples in GSE58720 were obtained from mice subjected to 90 min of MCAO followed by 3 h of reperfusion. Among the three datasets were analyzed with GEO2R. Differentially expressed genes (DEGs) were identified using the cutoff value of adjust. *p* (adj. *p*) < 0.05 and |log2 Foldchange|> 1.

### Viral particles construction

Adeno-associated viral (AAV) constructs carrying Iba1 promoter with shRHBDF2 (shRHBDF2^AAV9^) or shNC (shNC^AAV9^) sequence were packaged into recombinant AAV9 viral particles to achieve conditional knockdown of RHBDF2 in microglia for in vivo study. Groups of mice were given stereotaxic injections of either shRHBDF2^AAV9^ (2 µL, 10^13^ vg/mL) or shNC^AAV9^ into the cerebral cortex (from bregma: 0.25 mm posterior, 1.5 mm ventral, and 3 mm lateral). Downregulation of RHBDF2 expression in HMC3 cells was achieved with the recombinant lentiviral vectors (pLVX) carrying negative control (shNC^LV^) or shRHBDF2 (shRHBDF2^LV^) sequence.

### Animal model and treatment

All in vivo experiments were performed in accordance with approved animal protocols and guidelines established by the General Hospital of Northern Theater Command (No.2023–26). A total of 128 mice were analyzed in this study. The middle cerebral artery occlusion/reperfusion (MCAO/R) mouse model was established with reference to a previous study (Bertrand et al. 2017). The nylon monofilament (0.21 mm in diameter, #45–0400) was used to induce focal cerebral ischemia. The nylon monofilament was placed in the middle cerebral artery. Mice experienced same surgery without vessel occlusion served as sham. After 90 min, the nylon monofilament was removed, and the mice were allowed to recover for 24 h. Three weeks before MCAO surgery, shNC^AAV9^ or shRHBDF2^AAV9^ viral particles (2 µL, 1 × 10^13^ vg/mL) were intracerebroventricular injected into mice (from bregma: 0.25 mm posterior, 1.5 mm ventral, and 3 mm lateral). Once the mice regained consciousness, their neurological function was assessed according to the study of Longa et al*.* (Longa et al. 1989). 0, no noticeable neurological deficits; 1, contralateral forelimb flexion; 2, circling toward the paralyzed side when walking; 3, leaning toward the side opposite the lesion; and 4, inability to walk spontaneously. A score between 1 and 3 indicated successful modeling.

### 2,3,5-triphenyltetraolium chloride (TTC) staining

After reperfusion, the brains were dissected and sliced to five thick coronal sections. The prepared 1% PBS-diluted TTC staining solution was added, and the section were incubated for 15 min in the dark at 37 °C. The sections were then photographed.

### Fluro-Jade-C (FJC) staining

The degenerating neurons were detected with the FJC staining kit that purchased from Solarbio (#G3262, China). Briefly, the mouse brain tissue sections were prepared, dewaxed, and incubated with potassium permanganate solution for 10 min. FJC staining solution was prepared according to the manufacturer's instructions. After being incubated in FJC staining solution for 20 min, the sections were observed by fluorescence microscopy.

### Cell culture

Human microglia cell line HMC3 cells were purchased from iCell (China) and maintained in MEM medium (Solarbio, China) supplemented with 10% fetal bovine serum (FBS, Tianhang, China). Cells were cultured at 37 °C in a humidified atmosphere.

The combination of oxygen and glucose deprivation (OGD) and reoxygenation was used to mimic CIRI in vitro (Sun et al. 2018). HMC3 cells were maintained in glucose-free medium (Minimum Essential Medium, Procell, China) and placed in a hypoxic environment for 4 h. The cells were then replaced with normal medium under standard culture conditions for 24 h.

For RHBDF2 knockdown, HMC3 cells were treated with shNC^LV^ or shRHBDF2^LV^ viral particles for 72 h, followed by OGD/R treatment. STING agonist, 1 μM diABZI (#GC35855, GLPBIO, USA), was added to the medium during OGD/R treatment.

### Immunohistochemistry (IHC) staining

Paraffin-embedded brain sections were deparaffinized and dehydrated. Loss of antigenicity in sections was reversed with heated antigen retrieval solution for 10 min, and excess solution was blotted off. Peroxidase blocking was performed by incubating the sections for 15 min in 3% H_2_O_2_ solution. The sections were incubated with 1% bovine serum albumin (BSA, Sangon, China) for 15 min. The sections were incubated in primary antibody RHBDF2 (#AP13588A, Abcepta, China) overnight at 4 °C, and incubated with goat anti-rabbit IgG secondary antibody for 60 min at 37 °C. After being incubated with 100 μL DAB solution (#DAB-1031, MXB Biotechnology, China), the sections were counterstained with hematoxylin and photographed after mounting with neutral balsam.

### Immunofluorescence (IF) staining

The prepared brain tissue sections were incubated with antigen retrieval solution and kept at high temperature for 10 min. The cell coverslips were fixed with paraformaldehyde and incubated with Triton X-100. The coverslips and sections were then blocked with 1% BSA for 15 min at room temperature. The STING (#19851-1-AP, Proteintech, China) primary antibody was then used to incubate the slides overnight. The slides were washed thrice with PBS for 5 min each. The Cy3- preabsorbed secondary goat anti-rabbit (IgG) diluted in PBS was then used to incubate the slides at room temperature for 60 min. After being counterstained with DAPI and applied one drop of anti-fade mounting medium, the slides were then observed under the microscope.

For double IF staining experiments, the sections were heated in an antigen retrieval solution and cooled to room temperature. After blocking endogenous peroxidase activity, the sections were incubated with 1% BSA for 15 min. The sections were incubated in the mixture of primary antibodies overnight at 4 °C. Then, the sections were then incubated with secondary antibodies for 90 min. After being counterstained with DAPI and applied one drop of anti-fade mounting medium, the slides were then examined using the microscope. The following combinations and concentrations of antibodies were used: Iba-1 (#ab283319, 1:200, Abcam, UK), CD16 (#16559-1-AP, 1:100, Proteintech, China), CD206 (#18704-1-AP, 1:100, Proteintech, China).

### TUNEL-IF staining

Paraffin-embedded brain sections were deparaffinized and dehydrated. For permeabilization, the sections were incubated with 0.1% Triton X-100. After being incubated with antigen retrieval solution, the sections were incubated with TUNEL solution (Roche, Switzerland) for 60 min in the dark at 37 °C. The sections were then incubated with 1% BSA for 30 min in a humid environment. The primary antibody NeuN (#ab104224, Abcam, UK) was then used to incubate the slides overnight. The slides were then incubated with the secondary antibody for 60 min at room temperature. The slides were then observed under the microscope.

### Immunoprecipitation and western blot analysis

For immunoprecipitation, the protein sample was isolated with Native Lysis Buffer (Solarbio) containing protease inhibitor cocktail (Solarbio). The supernatants were incubated with anti‐STING antibody or anti‐IgG antibody conjugated AminoLink Coupling Resin (Thermo) for 2 h. After being incubated, the bead-bound proteins were dissociated and further analyzed using western blot analysis.

The protein sample of ischemic mouse cerebral tissues and HMC3 cells was homogenized with RIPA buffer (Solarbio) containing protease inhibitor cocktail (Solarbio) and phosphatase inhibitor cocktail (Solarbio). After treatment with ice-cold lysis buffer for half an hour, the lysates were centrifuged at 10000 g for 5 min at 4 °C. For the extraction of total mitochondrial proteins from HMC3 cells, the mitochondrial protein extraction kit (Solarbio, #EX1110) was utilized. Protein samples were separated by SDS-PAGE and transferred to polyvinylidene difluoride (PVDF, Millipore, USA) membranes. After being blocked with blocking buffer (Solarbio), the membranes were incubated with primary antibodies diluted in antibody dilution buffer (Solarbio) at 4 °C overnight. The membranes were incubated with secondary antibodies for 1 h at 37 °C, and the membranes were developed using ECL western blotting substrate (Solarbio). Primary antibodies were used as follows: RHBDF2 primary antibody (Abcepta, #AP13588A), STING primary antibody (Proteintech, #19851-1-AP), YTHDF1 primary antibody (Proteintech, #17479-1-AP), TBK1 primary antibody (Affinity, #DF7026), p-TBK1^Ser172^ primary antibody (Affinity, #AF8190), NF-kB p65 primary antibody (Affinity, #AF5006), p-NF-kB p65^Ser536^ primary antibody (Affinity, #AF2006), IRF3 primary antibody (Affinity, #DF6895), phosphorylated IRF3 (p-IRF3^Ser396^) primary antibody (Affinity, #AF2436), Arg1 primary antibody (Affinity, #DF6657), iNOS primary antibody (Affinity, #AF0199), Cytochrome C primary antibody (Affinity, # AF0146), and COX4 primary antibody (GeneTex, #GTX49132).

### Quantitative real-time PCR (qPCR)

RNA samples were extracted using TRIpure (BioTeke, China) and then reverse transcribed to cDNA using All-in-One First-Strand SuperMix (Magen Biotech, China). qPCR was performed using 2 × Taq PCR MasterMix and SYBR Green that were purchased from Solarbio. Primers for qPCR were as follows. Mus RHBDF2-F, AGCCCTCATCCTCGTGTC; Mus RHBDF2-R, CTGGTCTAGCTCGTACTTCTCA. Mus β-actin-F, GCCAGAGCAGTAATCTCCTTCT; Mus β-actin-R, AGTGTGACGTTGACATCCGTA. Homo RHBDF2-F, TGCCTCGTGTCTGTGGTCT; Homo RHBDF2-R, CGGTATGGGAGAAAGATGG. Homo YTHDF1-F, CAATGAGGCTCCGTGGTC; Homo YTHDF1-R, AAACAGCATCGTGCATAAAA. Homo β-actin-F, TCAGGGTGAGGATGCCTCTC; Homo β-actin-R, CTCGTCGTCGACAACGGCT.

### Cytokines determination

The levels of cytokines in brain tissue homogenates and cell supernatants were measured using ELISA kit that purchased from Multi Sciences Biotech Co., Ltd (China) according to the manufacturer's instructions. The concentrations of pro-inflammatory and anti-inflammatory cytokines including TNF-α (#EK282 for mouse, #EK182 for human), IL-10 (#EK210 for mouse, #EK110 for human), IL-1β (#EK201B for mouse, #EK101B for human), IL-6 (#EK106 for mouse), and IL-4 (#EK104 for human) in mouse brain samples and cell supernatants were measured with the corresponding ELISA kits.

### Flow cytometry

To detect the polarization of HMC3 cells, the cells were centrifuged (300 g for 5 min), washed, and resuspended. The resuspended cells (1 × 10^6^) were incubated with 5 μL CD16-PE (F1101602, Liankebio, China) or 4 μL CD206-FITC (FITC-65155, Proteintech Group, USA) antibody at 4 °C in darkness for 30 min. The stained cells were washed, resuspended, and read on the NovoCyte flow cytometer (Aglient, USA). After adding DCFH-DA, fluorescence microscopy was used for imaging, and flow cytometry was used to quantify the fluorescence intensity of 2,7-dichlorodihydrofluorescein (DCF), a detector of ROS.

### Methylated RNA immunoprecipitation (Me-RIP) PCR assay

The Me-RIP assay was performed using riboMeRIPTM m6A Transcriptome Profiling Kit according to manufacturer’s instructions. Total RNA (1 μg/μL) was fragmented into about 100 bp ~ 150 bp. For the preparation of Me-RIP beads, 25 μL magnetic beads A/G were incubated with 5 μg anti-m6A antibody in 250 μL IP buffer for 30 min at room temperature. IP reaction mixture (500 μL, containing fragmented RNA) was used to resuspend the prepared beads and incubated at 4 °C for 2 h. After being washed in IP buffer, the reaction mixture was eluted at 4 °C for 1 h with continuous shaking. The eluted RNA was further purified using the Magen Hipure Serum/plasma miRNA Kit. RNA m6A modification was then tested by qPCR assay. Primers for qPCR were as follows. Mus RHBDF2-5’UTR-F, CACAGCCCAACCGTCCA; Mus RHBDF2-5’UTR-R, AGGCAGAAGAGGCACCG. Mus RHBDF2-CDS-F, GGGCAAGCGACAAAACTG; Mus RHBDF2-CDS-R CCTGGAAGGATGGCACCT. Homo RHBDF2-CDS-F, AGCGAGACCTGGAGCGG; Homo RHBDF2-CDS-R, AGGGGCGGCCCTTGATC.

### RNA immunoprecipitation coupled with PCR

YTHDF1 RIP was performed using the EZ-Magna RIP™ RNA-Binding Protein Immunoprecipitation Kit (Millipore) according to the manufacturer’s protocol. For each reaction, 5 μg of anti-YTHDF1 antibody or the same amount of IgG was used.

### ATP measurements

Relative ATP content in HMC3 cells was measured with the ATP assay kit (Beyotime, #S0026) according to the manufacturer's instructions. Briefly, cells were treated with lysis solution and incubated. After the incubation, cells were centrifuged at 12,000 g for 5 min at 4 °C, and the supernatant was reserved. ATP assay working solution prepared and stand at room temperature for 5 min. ATP assay working solution (100 μL) and the prepared samples (20 μL) were mixed and detected immediately after 2 s interval. The samples corresponding luminescence were measured in microplate reader at 570 nm.

### RNA-sequencing

Total RNA from RHBDF2-knockdown HMC3 cells and its control cells were constructed and sequenced using the Illumina platform. The DEGs were generated using a cutoff value of|Log2Foldchange|> 1 and *p*. value < 0.05. The fragments per kilobase million (FPKM) values of each gene from different groups were used for gene set enrichment analysis (GSEA) by clusterProfiler. The protein–protein interaction (PPI) network of DEGs was constructed using the Search Tool for the Retrieval of Interacting Genes (STRING, https://string-db.org/). The top 10 hub genes were visualized using Cytoscape according to the identification of extracted PPI pairs. The relative abundance and proportion of immune cells in each sample was evaluated using CIBERSORT. Correlation between hub genes was generated using the corrplot package.

### Proximity ligation assay

Proximity Ligation Assay (PLA) to visualize protein–protein interactions was performed using the Duolink® In Situ Detection Reagents Red DUOLINK (#DUO92008) according to the manufacturer’s protocol. Briefly, after OGD/R treatment, cells were fixed for 15 min with 4% PFA and washed 3 times with PBS. Cells were then permeabilized using 0.1% Triton X-100 for 30 min and then blocked for 15 min at room temperature using 1% BSA. Cells were then incubated overnight at 4 °C in STING antibody (Proteintech, #66,680–1-Ig) or RHBDF2 antibody (Thermo, # PA5-121,462). The next day, cells were incubated in anti-mouse plus (#DUO92002) and anti-rabbit minus (#DUO92004) proximity ligation assay probes diluted 1:5 in the provided antibody diluent. Cells were then washed, and then the ligase was added to cells for 30 min at 37 °C. This step was followed by further washing with PBS, prior to addition of the polymerase (diluted in amplification buffer, 1:80) for 100 min at 37 °C. Subsequently, cells were washed using PBS, then DAPI was used for nuclear staining. All samples were analyzed using a confocal microscope.

### Statistical analyses

Dates are presented as mean ± SD. Two-tailed Student’s t-test between two groups. Experiments containing more than two groups were analyzed with one-way ANOVA, followed by Tukey's or Dunnett’s multiple comparison tests as requested. All these statistical analyses and graphic representations were obtained by using the GraphPad Prism software.

## Results

### Microglial-specific RHBDF2 knockdown reduced cerebral infarct and neuroinflammation in mice after cerebral ischemia–reperfusion injury

**Fig. 1:**
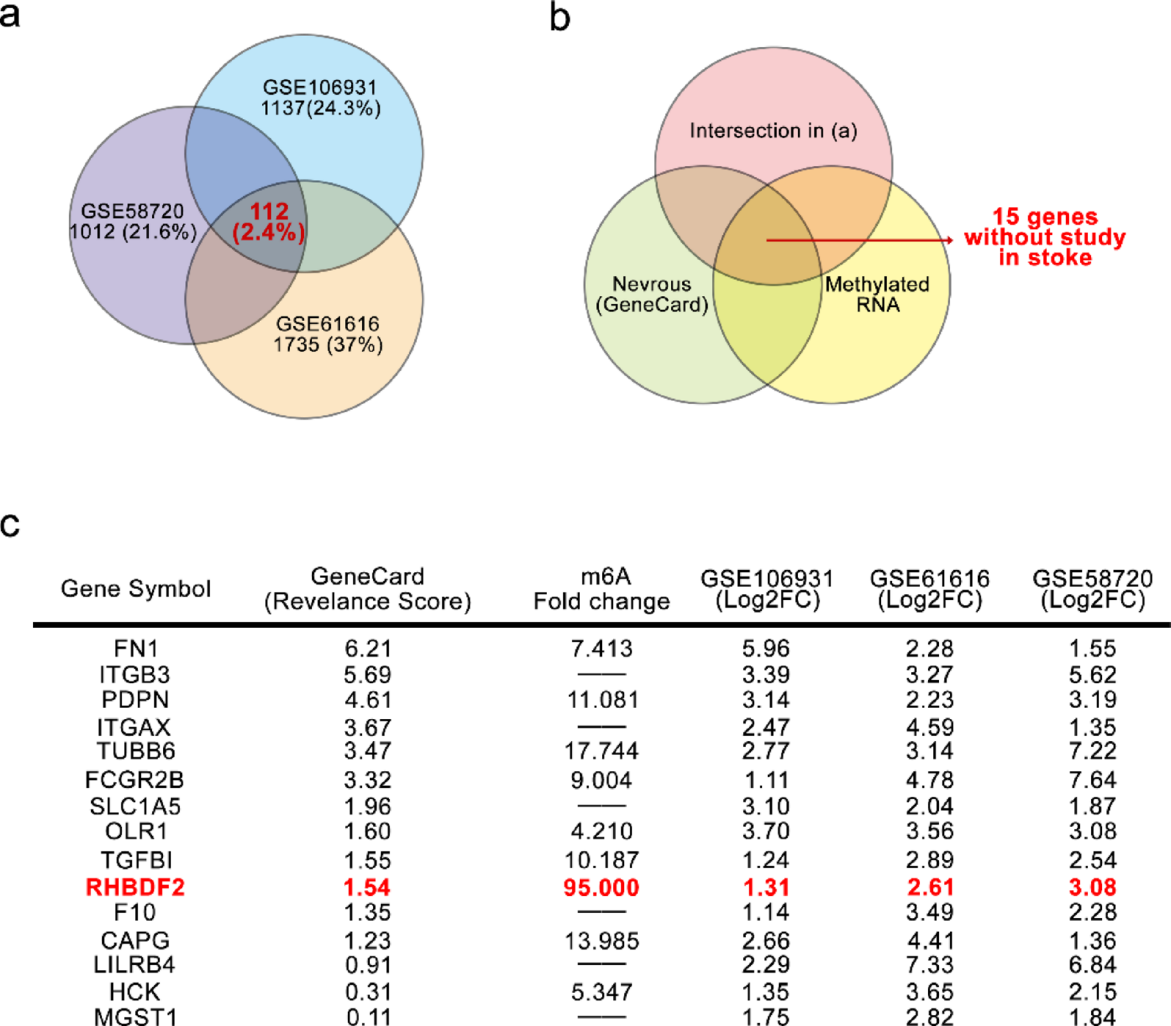
Targets related to microglial neuroinflammation in cerebral ischemia–reperfusion injury. **a** Venn diagram showing the overlap of differentially expressed genes (DEGs) among perivascular and meningeal macrophages from middle cerebral artery occlusion/reperfusion (MCAO/R) model animals (GSE106931, 1 h occlusion and 16 h reperfusion.), peri-infarct brain samples from MCAO/R model animals (GSE58720, 90 min occlusion and 24 h reperfusion), and brain tissue samples from MCAO model animals (GSE61616). **b** Venn diagram showing the overlap of differentially expressed genes (DEGs) among intersection in (a), neurons system related genes obtained from GeneCard, and differentially expressed m6A genes in MCAO/R rats obtained from previous study (20). **c** Table below showing the differential changes in 15 genes obtained from (b), details about relevance score, m6A expressed, and gene expression as shown in the table

Previous studies implicated that the aggravation of inflammatory response was regulated by microglia polarization in CIRI (Peng et al. 2024). To investigate the possible genes that functioned via microglia, using MCAO/R associated GEO datasets, the potential genes that were significantly altered in macroglia during MCAO/R (Fig. 1a). The occurrence of RNA m6A modification has been previously demonstrated in CIRI (Yu et al. 2023). Combined with the study of Yi et al*.* (2021), the significantly differentially expressed genes related to the nervous system and with m6A methylation, of which 15 genes have not been studied in stroke (Fig. 1b). As shown in Fig. 1c, RHBDF2 was identified as the most m6A modified gene in CIRI. The mRNA expression level of RHBDF2 was significantly upregulated in MCAO/R treated animals (Fig. 1c).

**Fig. 2:**
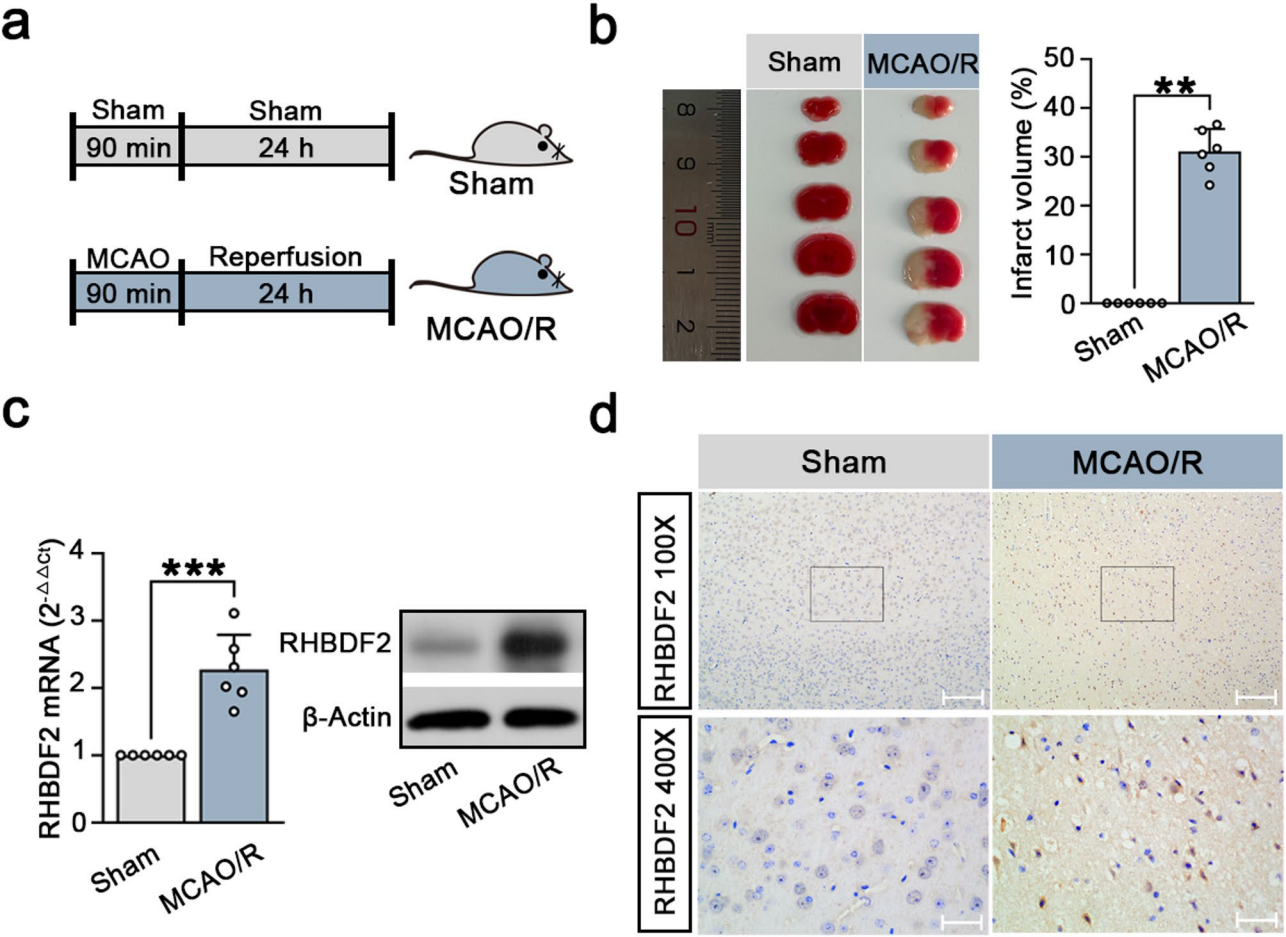
RHBDF2 expression was upregulated in cerebral ischemia–reperfusion injured mice. **a** Timeline schematic of MCAO/R surgery-induced cerebral ischemia and reperfusion injury (CIRI). **b** 2,3,5-triphenyltetrazolium chloride (TTC) staining images (Left). The quantitative analysis of infarct volume in mouse brain sections (Right, N = 6). **c** RHBDF2 mRNA in ischemic penumbra of mouse cerebral cortex sections (Left, N = 6). Representative immunoblots of RHBDF2 in the ischemic penumbra of mouse cerebral cortex sections (Right). **d** Images of RHBDF2 immunohistochemical staining in ischemic penumbra of mouse cerebral cortex sections. 100 × magnification, Scale bars: 200 µm; 400 × magnification, Scale bars: 50 µm. Data are presented as mean ± SD. **p < 0.01 and ***p < 0.001

Then, RHBDF2 expression was detected in CIRI using the MCAO/R mouse model as illustrated in Fig. 2a. MCAO/R mice had a larger ischemic volume than sham mice (Fig. 2b). Markedly increased RHBDF2 expression was observed in ischemic penumbra after MCAO surgery (Fig. 2c, d, S2a).

**Fig. 3:**
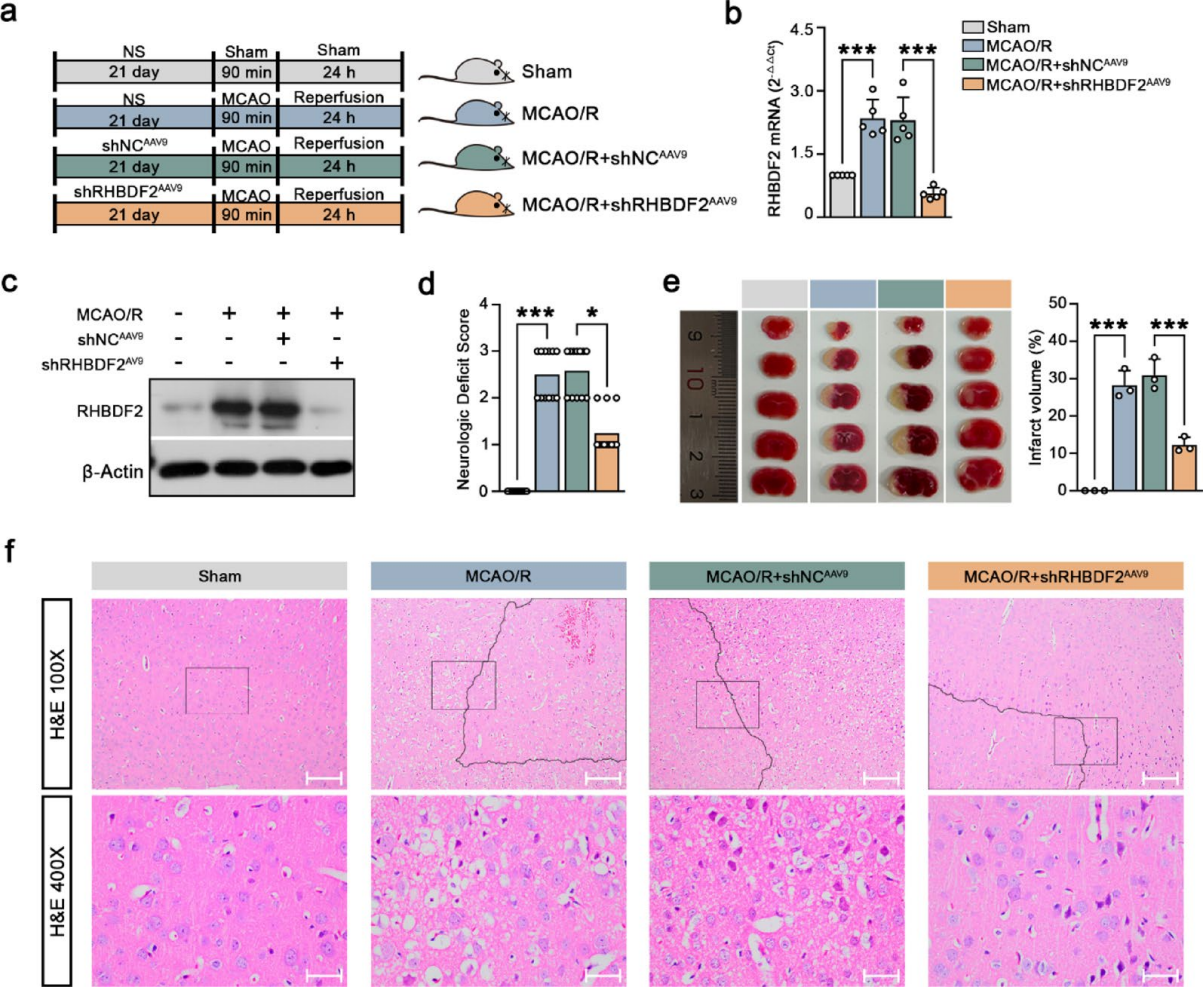
Microglial-specific RHBDF2 knockdown alleviated cerebral ischemia–reperfusion injury in MCAO/R mice. **a** Schematic diagram of MCAO/R-induced mouse model treated with RHBDF2 knockdown adenovirus (shRHBDF2^AAV9^) or negative control adenovirus (shNC^AAV9^). **b** RHBDF2 mRNA levels in ischemic penumbra of mouse cerebral cortex (N = 5). **c** Quantitative analysis of RHBDF2 protein expression in ischemic penumbra of mouse cerebral cortex (N = 5)*.* **d** The impact of RHBDF2 knockdown on mouse neurologic deficit (N = 11). **e** Representative TTC staining images in the cerebral infarct volume of MCAO/R mice (Left) and quantitation (Right, N = 3). **f** Representative results of hematoxylin–eosin staining in ischemic penumbra of mouse cerebral cortex sections. The lines indicate the ischemia boundary. 100 × magnification, Scale bars: 200 µm; 400 × magnification, Scale bars: 50 µm. Data are presented as mean ± SD. *p < 0.05 and ***p < 0.001

**Fig. 4:**
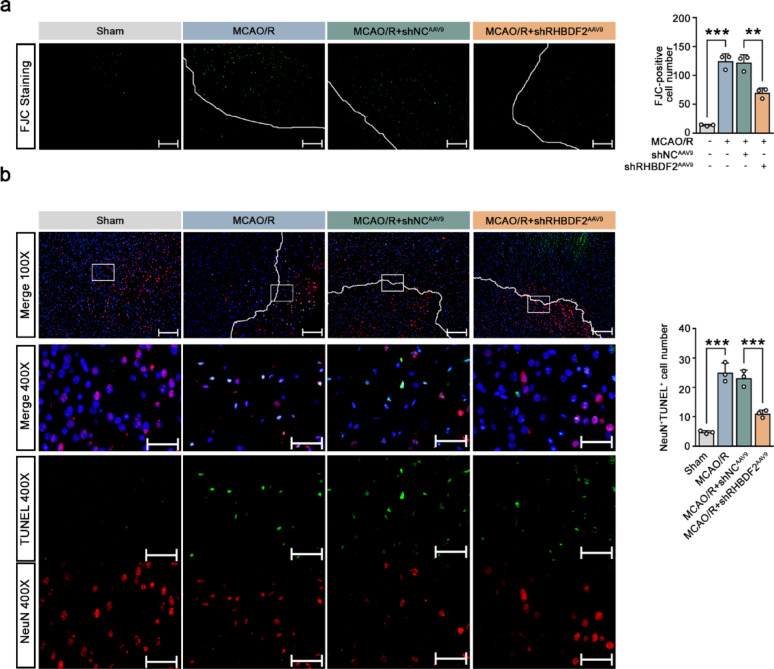
Microglial-specific RHBDF2 knockdown decreased neuron death in MCAO/R mice. **a** Representative images of FJC staining in ischemic penumbra of mouse cerebral cortex sections (Left). Quantification of FJC-positive cells (Right, N = 3). The lines indicate the ischemia boundary. 100 × magnification, Scale bars: 200 µm. **b** Double immunofluorescence staining for NeuN and TUNEL in the mouse ischemic penumbra sections (Left). Quantification of NeuN and TUNEL double positive cells (Right, N = 3). The lines indicate the ischemia boundary. 100 × magnification, Scale bars: 200 µm; 400 × magnification, Scale bars: 50 µm. Data are presented as mean ± SD. **p < 0.01 and ***p < 0.001

To investigate the role of microglial RHBDF2 in CIRI, microglia-specific RHBDF2 knockdown was performed using AAV constructs carrying Iba1 promoter in vivo (Fig. 3a). The downregulation of RHBDF2 expression was confirmed in the ischemic penumbra of MCAO/R-treated mouse (Fig. 3b, c, S2b). Microglial-specific RHBDF2 knockdown alleviated neurological deficits and decreased infarction volumes (Fig. 3d, e). Necrosis was reduced in the ischemic penumbra of MCAO/R mice with microglial-specific RHBDF2 knockdown (Fig. 3f). FJC-positive cells were significantly increased in the ischemic penumbra of mouse cerebral cortex sections after MCAO/R, and RHDBF2 knockdown markedly decreased FJC-positive cell number (Fig. 4a). The number of TUNEL and NeuN double-positive cells was also significantly downregulated in microglial RHBDF2 knockdown group (Fig. 4b). These data suggest that RHBDF2 expression was significantly upregulated in CIRI and probably functioned in microglia.

### Microglial-specific RHBDF2 knockdown modulated microglial polarization towards anti-inflammatory M2 phenotype in mice after cerebral ischemia–reperfusion injury

**Fig. 5:**
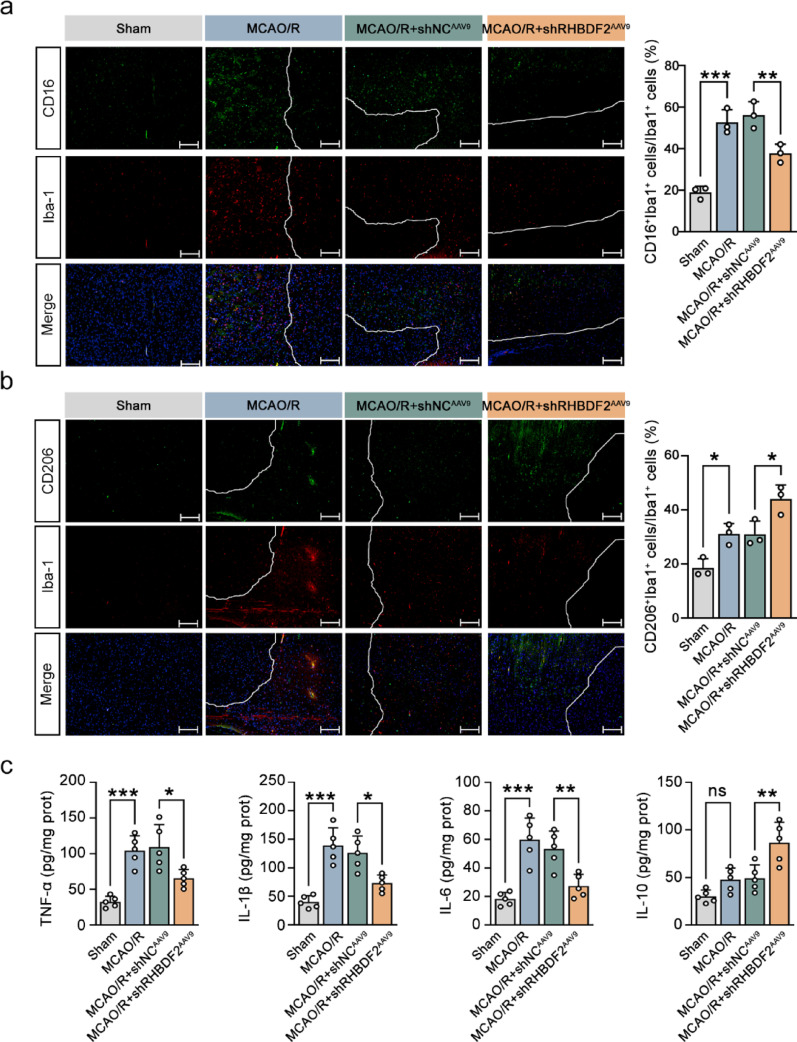
Microglial-specific RHBDF2 knockdown modulated microglial polarization towards anti-inflammatory M2 phenotype in MCAO/R mice. **a** Double immunofluorescence staining for CD16 and Iba-1 in the ischemic penumbra of mouse cerebral cortex sections (Left). Quantification of CD16 and Iba-1 double positive cells (Right, N = 3). The lines indicate the ischemia boundary. 100 × magnification, Scale bars: 200 µm. **b** Double immunofluorescence staining for CD206 and Iba-1 in the ischemic penumbra of mouse cerebral cortex sections (Left). Quantification of CD206 and Iba-1 double positive cells (Right, N = 3). The lines indicate the ischemia boundary. 100 × magnification, Scale bars: 200 µm. **c** TNF-α, IL-1β, IL-6, and IL-10 levels in the ischemic penumbra of mouse cerebral cortex sections (N = 5). Data are presented as mean ± SD. ns = not significant,*p < 0.05, **p < 0.01, and ***p < 0.001

The above results indicated the protective role of microglial RHBDF2 knockdown against CIRI in vivo. The transition of M1 microglia to M2 microglia has been demonstrated as an effective step to alleviate CIRI (Xu et al. 2021). Further experiments were performed to investigate the role of RHBDF2 in microglial polarization. As shown in Fig. 5a, the percentage of microglia expressing the M1 marker CD16 was upregulated in MCAO/R surgery mice, whereas microglial RHBDF2 knockdown decreased the percentage of M1 microglia. Accordingly, M2 microglia was enriched in the cerebral infarction tissues of MCAO/R mice, and the percentage of M2 microglia was further increased with RHBDF2 knockdown (Fig. 5b). RHBDF2 knockdown downregulated the levels of pro-inflammatory cytokines IL-6, TNF-α, and IL-1β, accompanied by the upregulated anti-inflammatory cytokine IL-10 levels (Fig. 5c).

**Fig. 6:**
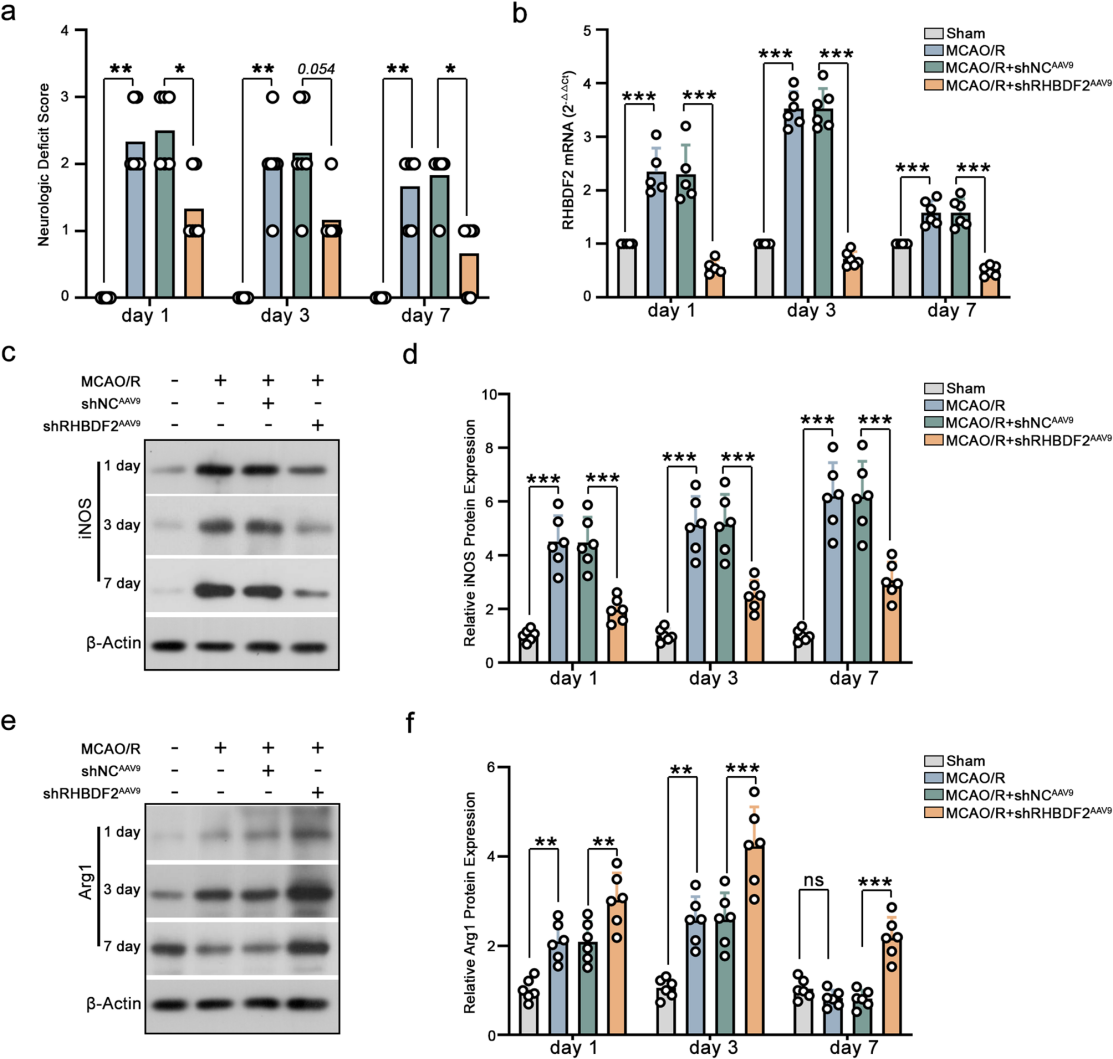
Effects of microglial RHBDF2 knockdown on long-term recovery of neurological function after MCAO/R in mice. **a** The impact of microglial RHBDF2 knockdown on mouse neurologic deficit at day 1 (N=5), 3, and 7 after MCAO (N = 6). **b** RHBDF2 mRNA levels in ischemic penumbra of mouse cerebral cortex sections at day 1, 3, and 7 after MCAO (N = 6). **c** Representative immunoblots of iNOS in the ischemic penumbra of mouse cerebral cortex sections at day 1, 3, and 7 after MCAO/R. **d** Quantitative analysis of iNOS protein expression in the ischemic penumbra of mouse cerebral cortex sections at day 1, 3, and 7 after MCAO (N = 6). **e** Representative immunoblots of Arg1 in the ischemic penumbra of mouse cerebral cortex sections at day 1, 3, and 7 after MCAO. f, Quantitative analysis of Arg1 protein expression in the ischemic penumbra of mouse cerebral cortex sections at day 1, 3, and 7 after MCAO (N = 6). Data are presented as mean ± SD. ns = not significant, *p < 0.05, **p < 0.01, and ***p < 0.001

A previous study indicated that the acute phase occurs on Days 1–3 after stroke onset, the subacute phase occurs on Days 3–8, and the chronic phase occurs after Day 8 (Jocher, et al. 2025). The M2 phagocyte response has been reported to be transient, phasing out within seven days after CIRI (Xu et al. 2020). This indicates that M2 microglia function in the acute and subacute phases of CIRI. Therefore, the role of RHBDF2 knockdown in chronic stroke recovery was investigated. As shown in Fig. 6A, RHBDF2 knockdown in microglia alleviated mouse neurological deficits on day 7 after MCAO. RHBDF2 mRNA expression peaked at three days after CIRI (Fig. 6b). The inhibitory role of RHBDF2 knockdown in decreasing iNOS expression continued for seven days (Fig. 6c, d). Interestingly, in MCAO/R mice, Arg1 expression peaked on day 3 after CIRI and was lower than in sham mice on day 7 after CIRI. Expression of Arg1 in the RHBDF2 knockdown group was lower on day 7 than on day 3 of reperfusion after MCAO surgery (Fig. 6e, f). The above results demonstrated that knockdown of RHBDF2 in microglia might suppress neuroinflammation by promoting M2 polarization in mice during acute and subacute phase of CIRI.

### Microglial-specific RHBDF2 knockdown suppressed the activation of STING signaling in vivo

**Fig. 7:**
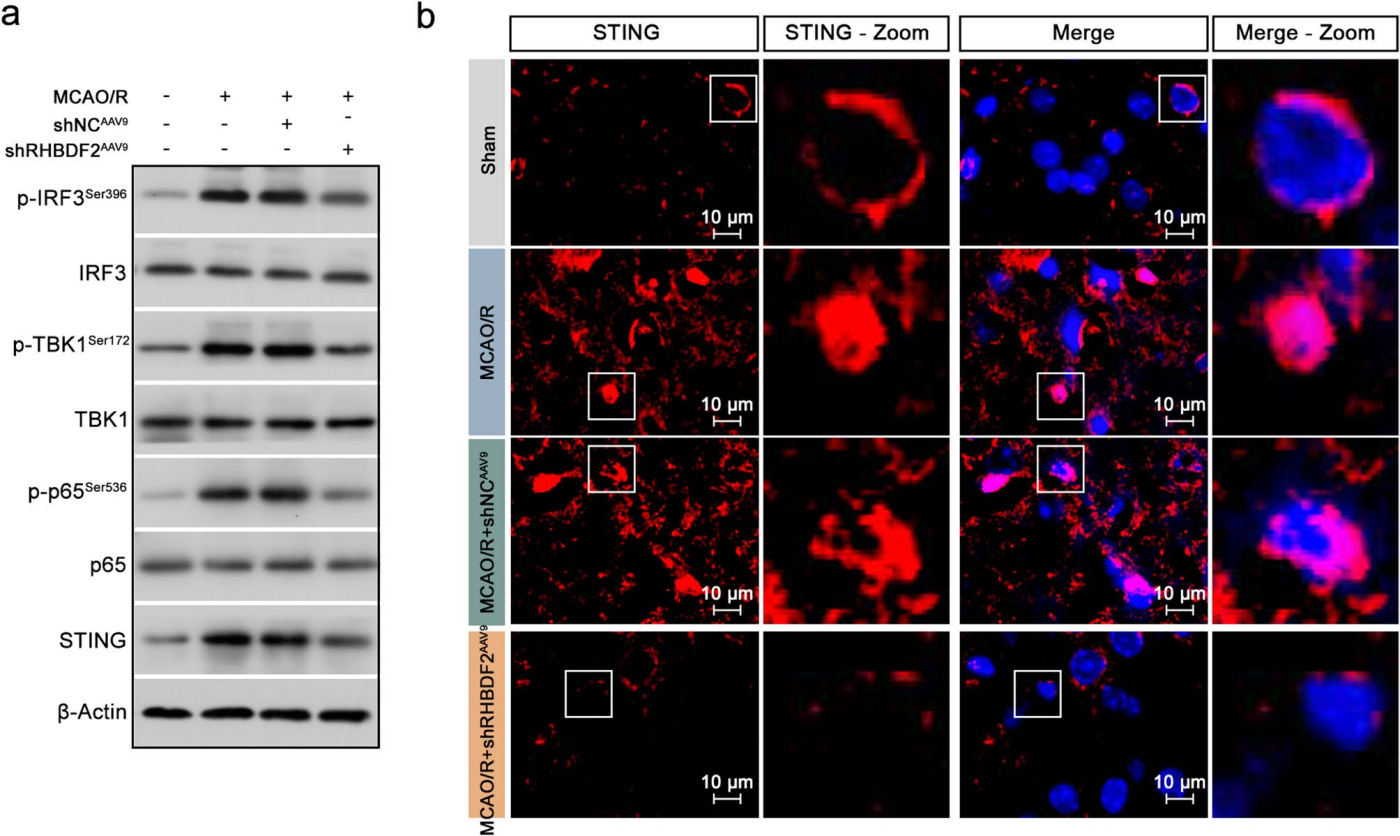
Microglial-specific RHBDF2 knockdown suppressed the activation of STING-TBK1 signaling pathway in vivo. **a** Representative immunoblots of phosphorylated IRF3 (p-IRF3^Ser396^), IRF3, p-TBK1^Ser172^, TBK1, p-p65^Ser536^, p65, and STING in the ischemic penumbra of mouse cerebral cortex sections. **b** Representative IF staining images of STING in ischemic penumbra of MCAO/R mouse cerebral cortex sections

It has been shown that the protein expression of STING could be regulated by RHBDF2 (Zhao et al. 2023; Luo et al. 2016), and the STING signaling pathway was involved in CIRI-induced microglial activation and neuroinflammation (Kong et al. 2022). The inhibition of microglial STING pathway ameliorated CIRI (Liao et al. 2020). Western blot analysis showed the upregulation of STING expression and phosphorylation of p65, TBK1, and IRF3 in the ischemic penumbra of cerebral cortex sections from MCAO/R mouse. Microglial RHBDF2 knockdown decreased STING expression and inhibited the activation of the downstream effectors (Fig. 7a, S2c). The increase in STING levels in the ischemic penumbra of MCAO/R mouse cerebral cortex sections and the downregulation induced by microglial RHBDF2 knockdown were also confirmed by IF staining (Fig. 7b), Taken together, we suggested that microglial RHBDF2 knockdown suppressed neuroinflammation with the inhibition of STING signaling pathway in vivo after CIRI.

### RHBDF2 knockdown promoted M2 microglial polarization in vitro after OGD/R

**Fig. 8:**
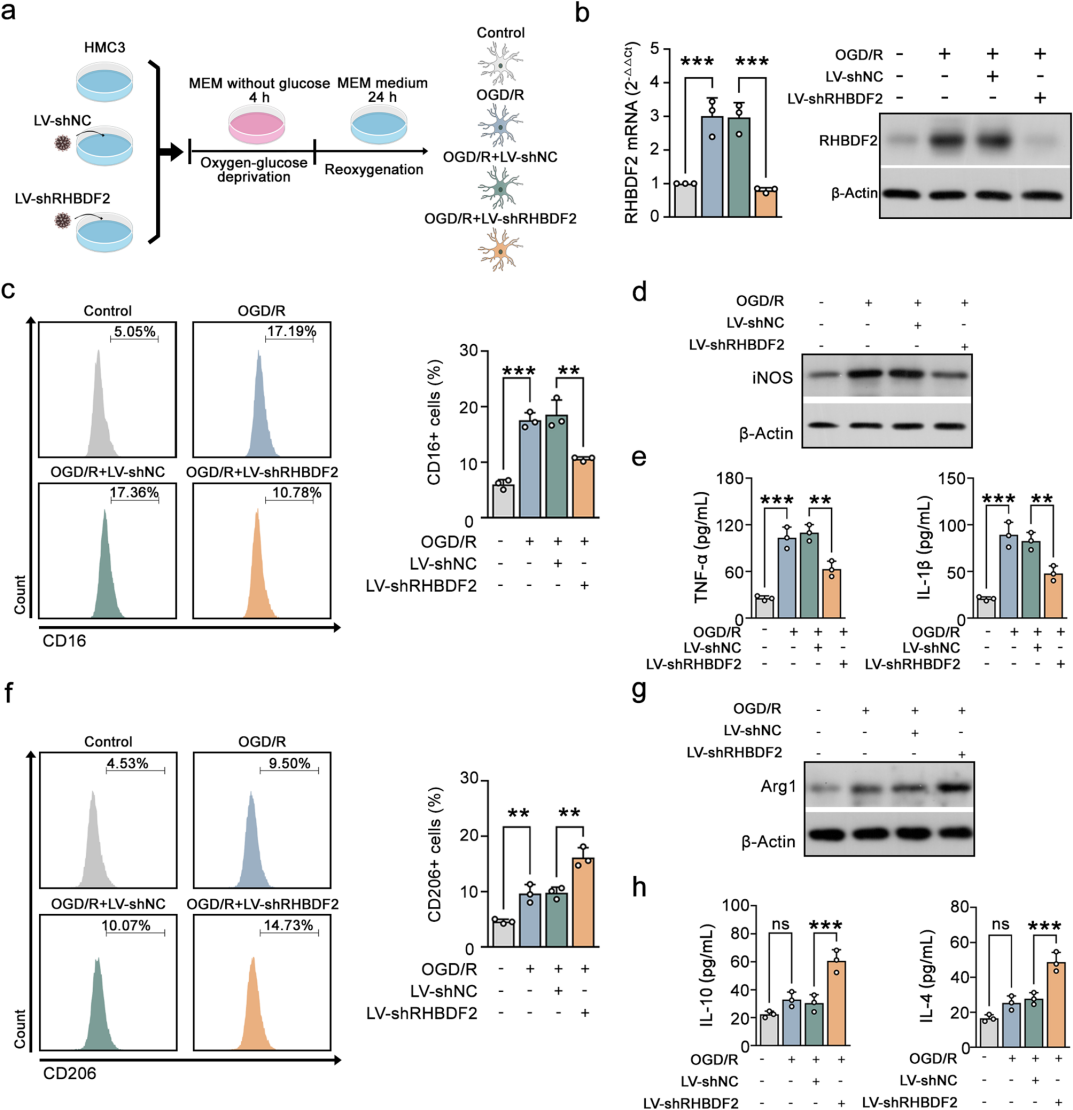
RHBDF2 knockdown promoted microglia M2 polarization in vitro after OGD/R. **a** Schematic of HMC3 cells with oxygen glucose deprivation/reoxygenation (OGD/R) treatment and lentivirus infection. **b** RHBDF2 mRNA in HMC3 cells (Left, N = 3). Representative immunoblots of RHBDF2 in HMC3 cells (Right). **c** Representative flow cytometry images (Left) and quantification analysis of CD16-positive HMC3 cells (Right, N = 3). **d** Representative immunoblots of iNOS in HMC3 cells. **e** TNF-α and IL-1β levels in the supernatant of HMC3 cells (N = 3). **f** Representative flow cytometry images (Left) and quantification analysis of CD206-positive HMC3 cells (Right, N = 3). **g** Representative immunoblots of Arg1 in HMC3 cells. **h** IL-4 and IL-10 levels in the supernatant of HMC3 cells (N = 3). Data are presented as mean ± SD. ns = not significant, **p < 0.01, and ***p < 0.001

To validate that RHBDF2 played the critical role in neuroinflammation via microglia polarization during CIRI, OGD/R-treated HMC3 cells was used to mimic CIRI in vitro (Fig. 8a). RHBDF2 protein expression in HMC3 cells were increased upon OGD/R treatment, and the knockdown efficiency was shown in Fig. 8b (Figure S3a). RHBDF2 downregulation decreased the percentage of CD16-positive cells in HMC3 cells after OGD/R (Fig. 8c), accompanied with the decreased iNOS protein expression (Fig. 8d, S3b). In addition, the production of proinflammatory cytokines (TNF-α and IL-1β) was dampened by RHBDF2 downregulation (Fig. 8e). Correspondingly, flow cytometry results showed that RHBDF2 knockdown significantly increased the percentage of M2 phenotype microglia (Fig. 8f). The expression of Arg1 was upregulated with RHBDF2 knockdown in OGD/R-treated HMC3 cells (Fig. 8g, S3c). As expected, anti-inflammatory cytokines were increased in the supernatant of HMC3 cells with RHBDF2 knockdown (Fig. 8h).

Microglial metabolic reprogramming has been shown to function in microglia polarization and neuroinflammation. Sun et al*.* have reported that elevated microglial oxidative phosphorylation stimulated brain remodeling and cognitive recovery after stroke in mice (Song et al. 2022). The study of Winther et al*.* reported that the inhibition of M1-associated mitochondrial oxidative phosphorylation was an important factor in preventing the M1 to M2 transition (Bossche et al. 2016). In RHBDF2 high expression Alzheimer's patients, the inhibition of oxidative phosphorylation was shown using RNA-seq (Zhang et al. 2024). The mitochondrion cytochrome c expression was decreased in OGD/R-treated HMC3 cells, and RRHBDF2 knockdown increased mitochondrion cytochrome c expression (Figure S1a). The production of ATP was decreased in HMC3 cell upon OGD/R stimulation and increased in RHBDF2-downregulated HMC3 cells (Figure S1b). Furthermore, in HMC3 cells, OGD/R-induced increase ROS level was reversed by RHBDF2 knockdown (Figure S1c). Above results indicated that RHBDF2 knockdown in microglia reversed OGD/R treatment-induced inhibition of oxidative phosphorylation. Collectively, above results suggested that RHBDF2 knockdown promoted M2 microglial polarization after CIRI.

### Activation of STING abrogated the effects of RHBDF2 knockdown on microglia polarization

**Fig. 9:**
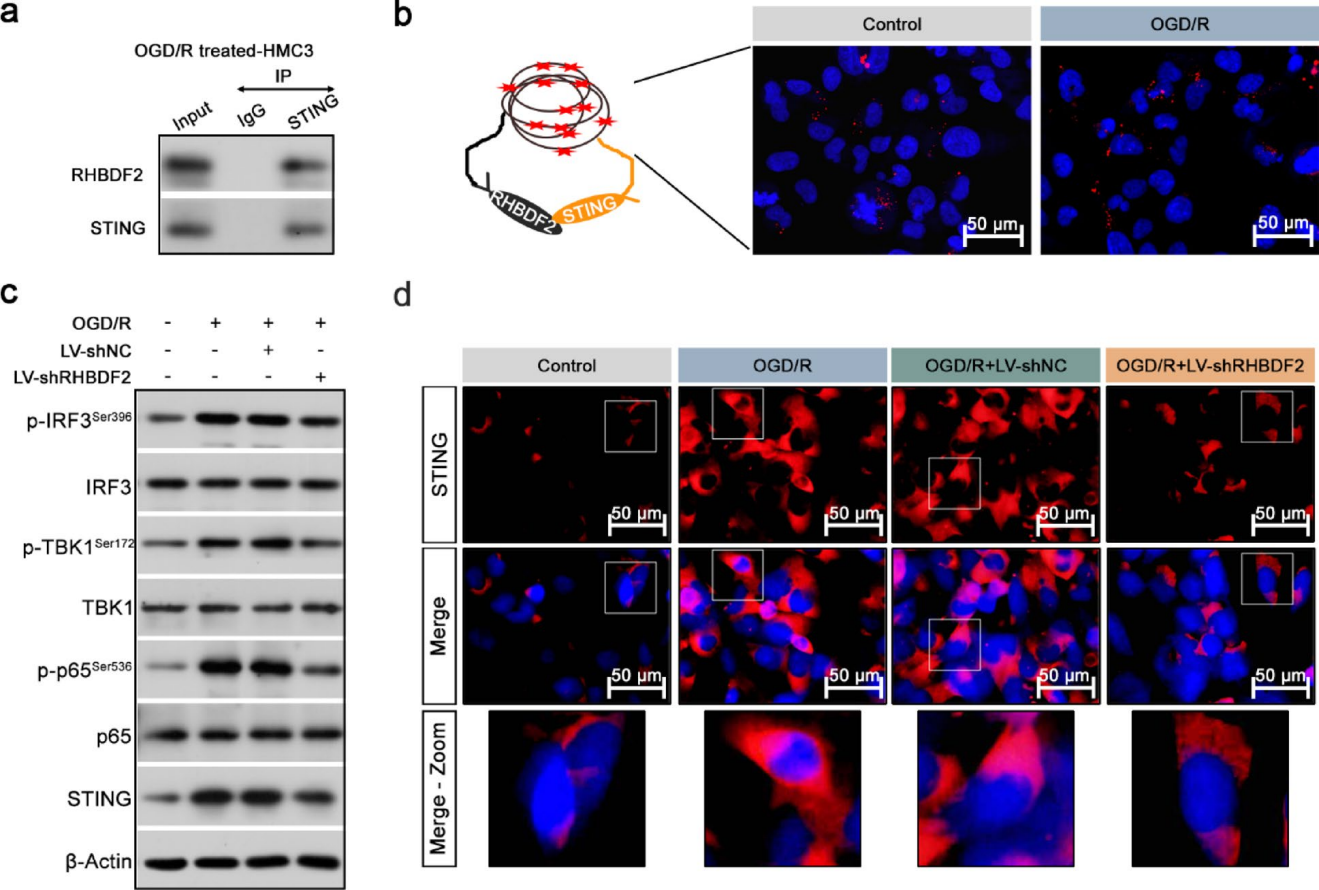
RHBDF2 promoted the activation of the STING-TBK1 signaling pathway in OGD/R-treated HMC3 cells. **a** Co-IP assay detected the interaction between RHBDF2 and STING in OGD/R-treated HMC3 cell. **b** Proximity ligation assay showing an interaction between STING and RHBDF2 in HMC3 cell. **c** Representative immunoblots of p-IRF3^Ser396^, IRF3, p-TBK1^Ser172^, TBK1, p-p65^Ser536^, p65, and STING in HMC3 cells. d, Representative IF staining images of STING in HMC3 cells

RHBDF2 has been shown to regulate the stability of STING, thereby participating in innate immunity (Luo and Shu 2017). With the performance of co-immunoprecipitation (Co-IP) analysis, the protein interaction between STING and RHBDF2 in HMC3 cells upon OGD/R treatment was confirmed (Fig. 9a). A proximity ligation assay, which is able to detect proteins only when in close proximity to each other was performed in OGD/R-treated HMC3 cells. The results showed proximity ligation assay dots in OGD/R-treated HMC3 cells and control HMC3 cells (Fig. 9b). The increased protein expression of STING and the phosphorylation of p65, TBK1, and IRF3 in HMC3 cells, triggered by OGD/R treatment, were reduced with RHBDF2 knockdown (Fig. 9c, S3d). RHBDF2 knockdown downregulated the increased STING expression during CIRI, which was also observed by IF staining, as shown in Fig. 9d.

**Fig. 10:**
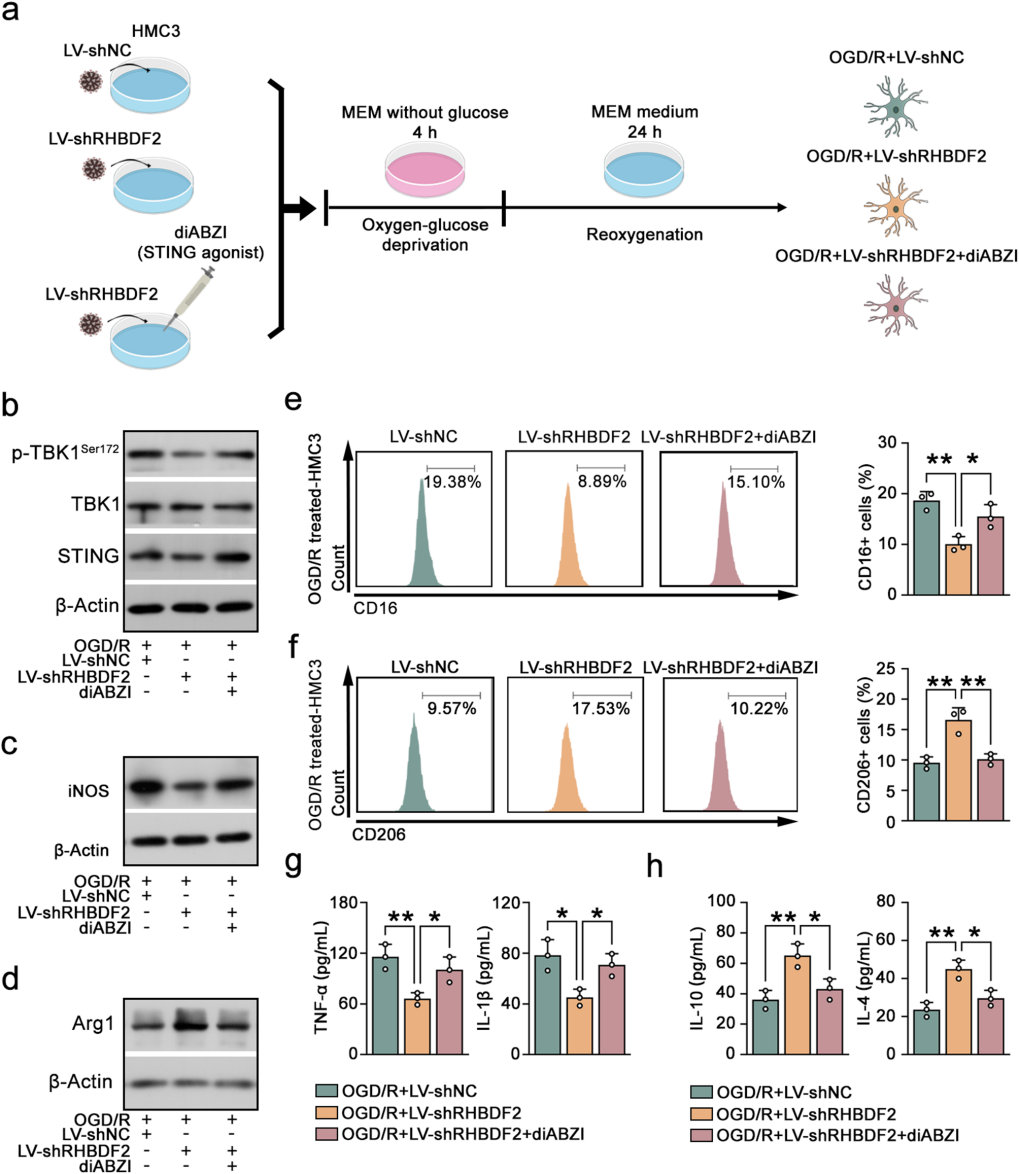
Activation of STING abrogated the effects of RHBDF2 knockdown on microglia polarization. **a** Schematic of OGD/R-stimulated HMC3 cells with STING agonist (diABZI) administration. **b** Representative immunoblots of p-TBK1^Ser172^, TBK1, and STING in HMC3 cells with diABZI treatment. **c** Representative immunoblots of iNOS in HMC3 cells with diABZI treatment. **d** Representative immunoblots of Arg1 in HMC3 cells with diABZI treatment. **e** Representative flow cytometry (Left) images and quantification analysis of CD16-positive HMC3 cells with diABZI treatment (Right, N = 3). **f** Representative flow cytometry images (Left) and quantification analysis of CD206-positive HMC3 cells with diABZI treatment (Right, N = 3). **g** TNF-α and IL-1β levels in the supernatant of HMC3 cells with diABZI treatment (N = 3). **h** IL-10 and IL-4 levels in the supernatant of HMC3 cells with diABZI treatment (N = 3). Data are presented as mean ± SD. *p < 0.05 and **p < 0.01

To further confirm that RHBDF2 affects microglia-induced neuroinflammation via the STING pathway in CIRI, the specific STING agonist (diABZI) was used in HMC3 cells with RHBDF2 knockdown (Fig. 10a). diABZI treatment reversed the decreased protein expression of phosphorylated TBK1 (p-TBK1) and STING that triggered by RHBDF2 knockdown (Fig. 10b, S3e). Administration of diABZI increased iNOS protein expression and decreased Arg1 protein expression in OGD/R treated-HMC3 cells (Fig. 10c, d, S3f-S3g). Flow cytometry showed that the addition of diABZI upregulated the percentage of CD16-positive cells and suppressed the percentage of CD206-postive cells (Fig. 8e, f). The secretion of pro-inflammation cytokines was markedly elevated, while the secretion of anti-inflammation cytokines was significantly decreased (Fig. 8g, h). In essence, our findings indicated that RHBDF2 played a pivotal role in microglial transition, at least in part, by promoting STING protein stability, thereby activating the STING pathway.

### YTHDF1 promoted RHBDF2 translation in m6A-dependent manner

**Fig. 11:**
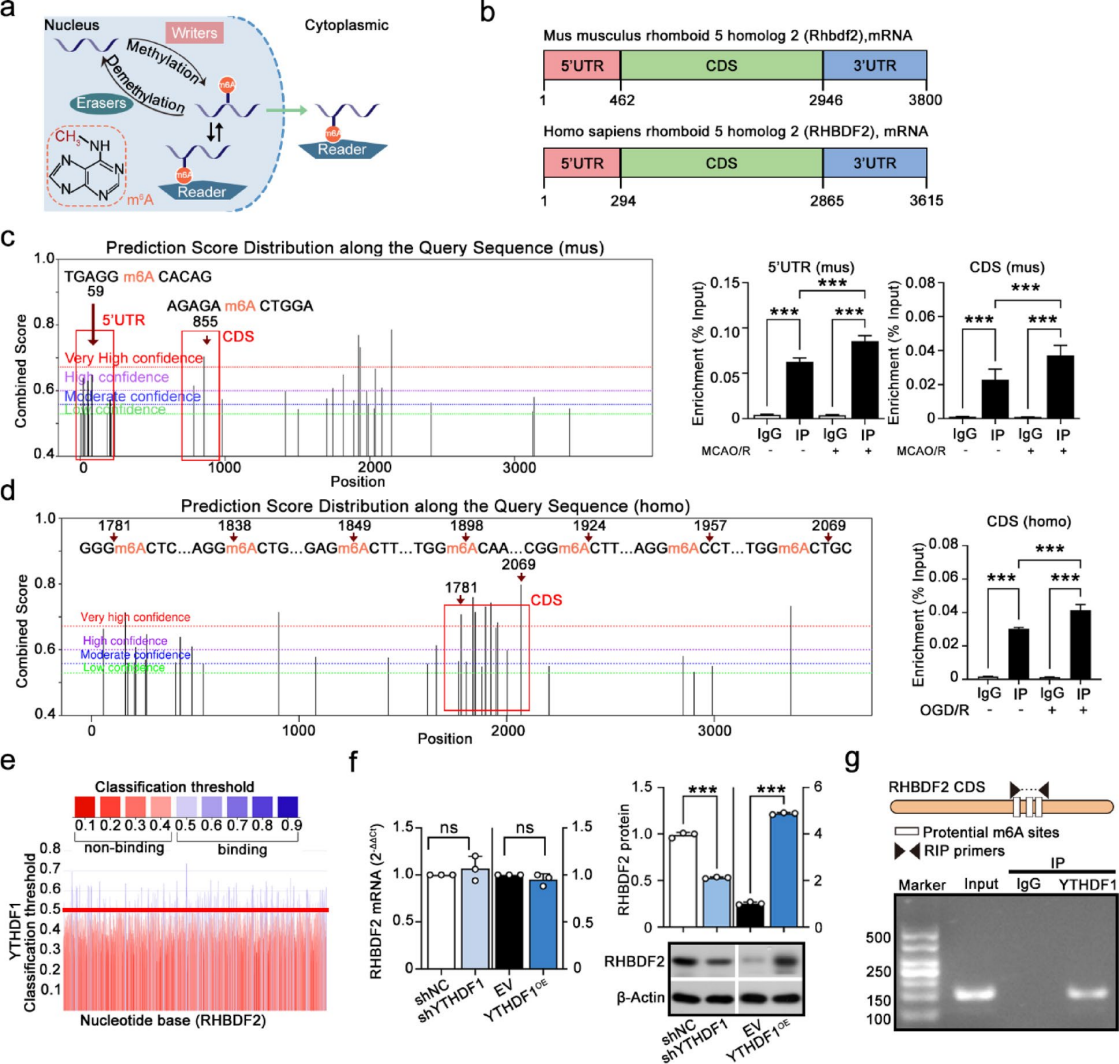
YTHDF1 promoted RHBDF2 translation in an m6A-dependent manner. **a**, Schematic of m6A modification mechanism. **b** Schematic showing the 5’UTR, 3’UTR and CDS position of mus sapiens Rhbdf2 and homo sapiens RHBDF2 gene. **c** Prediction of m6A modifications in mus sapiens RHBDF2 mRNA using SRAMP (http://www.cuilab.cn/sramp/). m6A levels of RHBDF2 in the ischemic penumbra of mouse cerebral cortex sections detected by Me-RIP PCR (N = 6). **d** Prediction of m6A modifications in homo sapiens RHBDF2 mRNA using SRAMP (http://www.cuilab.cn/sramp/). m6A levels of RHBDF2 in HMC3 cell determined by Me-RIP PCR (N = 3). **e** Prediction of binding sites between YTHDF1 protein and RHBDF2 mRNA by PRIdictor (http://www.rnainter.org/PRIdictor/). **f** RHBDF2 mRNA and protein expression in HMC3 cells with YTHDF1 knockdown or overexpression (N = 3). **g** Schematic representation of the potential YTHDF1 binding sites in RHBDF2 (up) and RIP assay confirms the enrichment of YTHDF1 in RHBDF2 mRNA in HMC3 cell (below). Data are presented as mean ± SD. ns = not significant, **p < 0.01, and ***p < 0.001

Previous study showed that RHBDF2 RNA methylated levels was involved in Alzheimer's disease (Jager et al. 2014). Therefore, further experiments were performed to investigated whether RHBDF2 m6A modification was involved in the molecular mechanism of CIRI. As shown in Fig. 11a, the schematic diagram illustrated the dynamic regulation of m6A methylation. Me-RIP PCR results showed that the m6A methylation level of RHBDF2 was significantly increased in mouse with MCAO/R surgery and OGD/R-stimulated HMC3 cell (Fig. 11b–d). YTHDF1 is the m6A reading protein that regulates mRNA translation (Ren et al. 2023), and YTHDF1-mediated gene expression has been shown to be involved in CIRI (Liu et al. 2024). With the analysis of PRIdictor database, YTHDF1 protein has a strong potential to bind to RHBDF2 mRNA (Fig. 11e). Based on the above considerations, we hypothesized that YTHDF1 might regulate microglial RHBDF2 expression in the m6A dependent manner. The upregulation or downregulation of YTHDF1 affected the protein expression of RHBDF2 (Fig. 11f). The results of RIP-PCR assay showed that YTHDF1 protein could bind to RHBDF2 mRNA (Fig. 11g). These results suggest that YTHDF1 might regulate microglial RHBDF2 expression in CIRI through post-transcriptional regulation.

### Investigation of the potential molecular mechanisms and hub genes upon RHBDF2 downregulation in cerebral ischemia–reperfusion injury

**Fig. 12:**
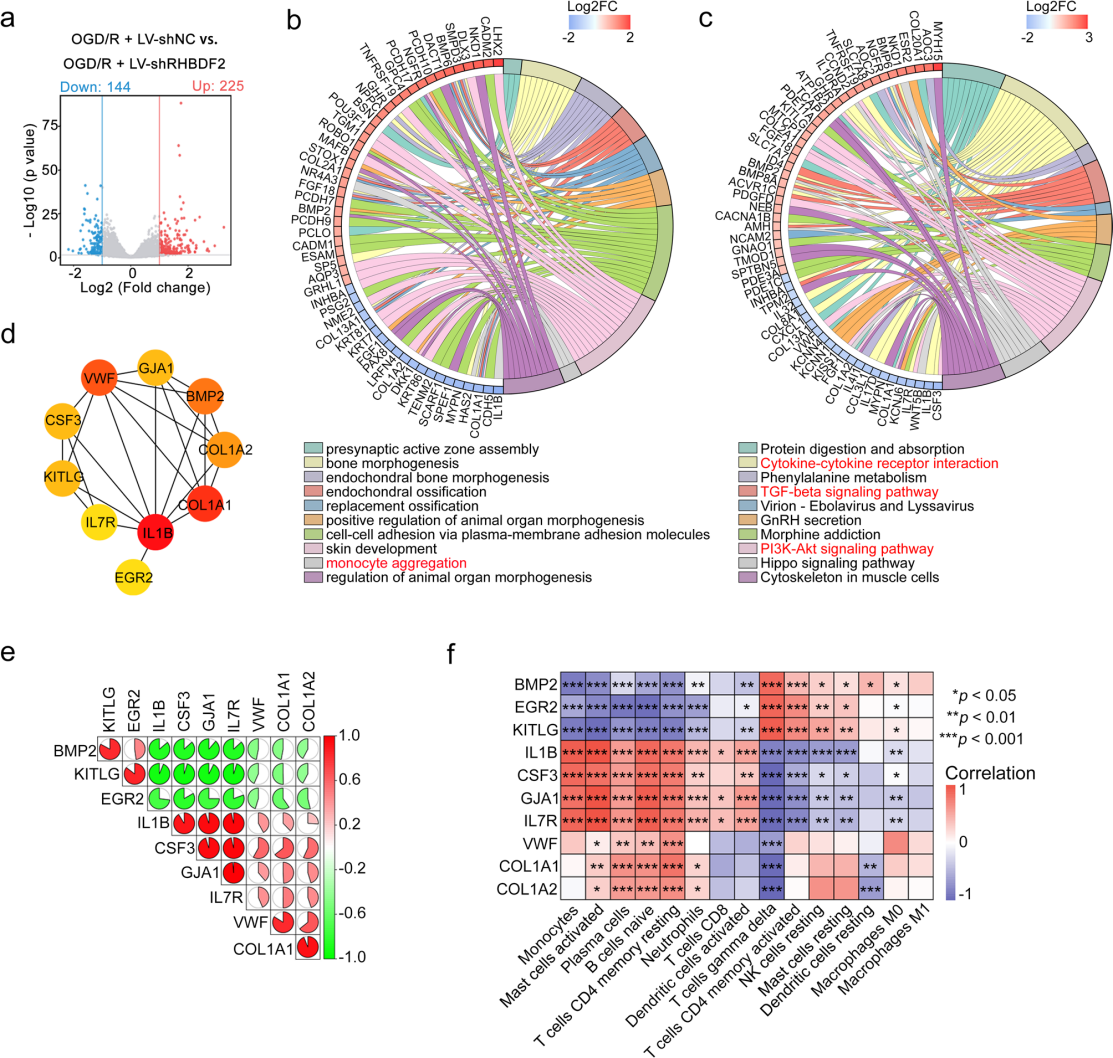
Investigation of the potential molecular mechanisms and hub genes upon RHBDF2 downregulation in OGD/R-treated HMC3 cells. **a** Volcano plot of DEGs between OGD/R + LV-shNC and OGD/R + LV-shRHBDF2 HMC3 cells. The x-axis is the Log2FoldChange value and the y-axis is -Log10 *p*-value. (|Log2FoldChange|> 1 and p. value < 0.05). **b** Chord diagram of Top 10 GO enrichment term and related genes (derived from DEGs). **c** Chord diagram of Top 10 KEGG enrichment term and related genes (derived from DEGs). **d** Protein interaction network of top 10 hub genes of DEGs. A deeper color of nodes represents a lower or higher fold change of degree. **e** The correlation analysis of 10 hub genes. **f** The correlation between hub genes and differential immune cells

RNA-seq was then performed in RHBDF2 knockdown HMC3 cells and its control cells, both subjected to OGD/R treatment. The volcano plot and heat map of DEGs were generated using a cutoff value of|log2 foldchange|> 1 and *p* value < 0.05 (Fig. 12a). GO enrichment found that RHBDF2 knockdown significantly downregulated the monocytes aggregation (Fig. 12b). The KEGG terms associated with inflammation, namely "cytokine-cytokine receptor interaction," "TGF-beta signaling pathway," and "PI3K-Akt signaling pathway," were found to be enriched in RHBDF2 knockdown HMC3 cells (Fig. 12c). Then, using the STRING database, the PPI network and evaluated potential hub genes were constructed (Fig. 12d). Interestingly, all 10 of these downstream possible genes are exoproteins. Combined with the specific expression of RHBDF2 in microglia of brain tissue suggests that RHBDF2 may crosstalk with other cells by influencing the secretion of downstream proteins.

IL1B was naturally enriched as a hub gene as it was an important regulator of the inflammatory response. As shown in the correlation between hub genes, EGR2 was negatively correlated with other hub genes (Fig. 12e). Immune cell infiltration showed that all hub genes were associated with mast cells, plasma cells, B cells, and T cells (Fig. 12f). To further explore the major biological functions and pathways affected by the 6 hub S-DEGs, single gene GSEA analysis was performed. The results showed that IL1B, CSF3, and BMP2 were associated with inflammatory response. COL1A1, COL1A2, VWF, IL7R were associated with cell adhesion and hematopoietic cells. Importantly, EGR2 was associated with brain and neuron development (Figure S4 and S5), suggesting EGR2 might be the downstream of RHBDF2 in nervous system. Combined with the GSEA analysis of hub genes, the possible downstream effectors of RHBDF2 at the mRNA level were revealed, which needs further research.

## Discussion

The acute inflammatory response and its resolution are crucial for the physiological repair process after injury, and excessive inflammation is associated with CIRI (Mo et al. 2020). Neuroinflammation progression is a complex, organized, and active process that requires intricate interactions between microglia and injured neurons. Previous studies have shown that excessive microglial activation leads to proinflammatory cytokine and chemokine release, which subsequently induces neuronal apoptosis (Kaewmool et al. 2020). However, little is known about brain factors controlling neuroinflammation regression and underlying mechanisms.

To investigate the possible genes and mechanisms of CIRI induced by microglia-mediated inflammation, the associated datasets were downloaded from the GEO database and nerve-related genes from the GeneCard database. m6A modification has been reported to be functioned in CIRI (Chang et al. 2022). Combined with the m6A transcriptome-wide map of CIRI from the study of Yi et al*.* (2021), the role of microglial RHBDF2 expression in CIRI was further investigated. We found for the first time that RHBDF2 is an important positive modulator of neuroinflammation after CIRI. In particular, the expression of RHBDF2 was significantly upregulated in microglia after ischemic stroke. In vivo, microglial RHBDF2 knockdown resulted in neuroprotection, as evidenced by decreased infarct size, neurological deficit scores, and cognition. The inhibition of RHBDF2 led to a significant reduction in the production of pro-inflammatory cytokines and chemokines both in vivo and in vitro. Therefore, our study suggests that RHBDF2 might be a promising therapeutic target for the treatment of ischemic stroke.

During the acute phase of CIRI, activated microglia recruits to the peri-infarct area and dynamically polarize into M1 or M2 phenotype (Wang et al. 2022). M1 microglia release inflammatory cytokines that can directly promote inflammation (Yunna et al. 2020). In contrast, M2 microglia secrete anti-inflammatory factors, such as IL-10 (Li et al. 2023a). Accumulating evidence suggests the inhibition of the M1 phenotype and stimulation of the M2 phenotype in the early stages of the ischemic penumbra has been shown to alleviate CIRI (Kanazawa et al. 2017). Previous study has demonstrated the inhibition of RHBDF2 alleviated neurological injury (Xu et al. 2020). Kim et al*.* have shown that RHBDF2 promoted macrophage proinflammatory mediator production, exacerbating lung ischemia–reperfusion injury (Kim et al. 2018b). Recent studies have reported that RHBDF2 expresses in the brain and mainly localized in microglia. In this study, microglial RHBDF2 knockdown shifted the polarization of microglia to the neuroprotective and tissue repair M2 phenotype in the ischemic penumbra, which improved the prognosis of mice subjected to MCAO/R. The direct effect of RHBDF2 on microglial polarization was confirmed in our study of OGD/R-stimulated HMC3. The similar results that RHBDF2 expression in macrophages induced phenotypic changes has been demonstrated by Zhou et al*.* (2020). Interestingly, M2 activation of microglia could be observed after OGD/R treatment, although it was not significant. Metabolic reprogramming plays an important role in microglia polarization. Elevated microglial oxidative phosphorylation stimulated brain remodeling and cognitive recovery after stroke in mice (Song et al. 2022). In this manuscript, RHBDF2 knockdown in microglia reversed OGD/R treatment-induced inhibition of oxidative phosphorylation. The above results suggested that RHBDF2 knockdown might have a protective effect in CIRI via suppressing neuroinflammation by promoting M2 microglia polarization.

STING is a dimeric transmembrane protein at the endoplasmic reticulum, and the immune role of the STING pathway in central nervous system disorders has grown in recent years (Yang et al. 2025; Sliter et al. 2018). In CIRI, STING has been verified to affect microglial polarization by influencing the downstream expression of TBK1/IRF3 (Kong et al. 2022). Similarly, our study indicated that the activation of STING signaling pathway in M1 phenotype microglia both in vivo and in vitro. RHBDF2 has been reported to mediate STING protein stability and increased STING expression in to DNA virus-induced innate immunity (Luo et al. 2016). With the performance of Co-IP experiments, there was a binding between the proteins of RHBDF2 and STING in microglia during CIRI. Furthermore, RHBDF2 knockdown inactivated the NF-κB signaling pathway during inflammation response (Lu et al. 2017). In our study, the administration of STING agonist reversed the effect of RHBDF2 knockdown in OGD/R-treated HMC3 cells. Our findings suggested that RHBDF2 increases STING protein stability, thereby activating the STING signaling pathway in microglia after CIRI. m6A modification has been elucidated to participate in the progression of CIRI (Chang et al. 2022). YTHDF1 is a typical m6A reader and participates in the regulation of CIRI by promoting target gene translation (Liu et al. 2024). In this study, for the first time, the connection between RHBDF2 and YTHDF1 was confirmed in OGD/R treated microglia. YTHDF1 promoted RHBDF2 translation in an m6A-dependent manner in microglia after cerebral ischemia and reperfusion injury.

RNA-seq was further used to learn more about the possible underlying mechanism of RHBDF2 in CIRI. Although this manuscript provides some evidence that RHBDF2-expressing microglia affect CIRI progression by regulating neuroinflammation, there are still some shortcomings. In normotensive rats, exogenous estrogen has been verified to increase stroke injury (Rusa et al. 1999). Therefore, to minimize the variables involved, adult male mice was used in our study. Previous study demonstrated that ischemic stroke exhibits significant sex differences in incidence, severity, and recovery. On the other hand, stroke mainly affects the elderly population and their prognosis is worse. Therefore, to fully demonstrated the role of microglia RHBDF2 in CIRI, further animal study was needed.

## Conclusion

In summary, our results indicated for the first time that microglial RHBDF2 knockdown could protect against CIRI by inhibiting microglial neuroinflammation. Our findings imply that RHBDF2 activated STING-TBK1 signaling pathway by the interaction between RHBDF2 and STING. Moreover, YTHDF1 positive regulated RHBDF2 protein expression in m6A-dependent manner. Therefore, our study demonstrated RHBDF2 as a promising strategy to alleviate CIRI, and possibly other types of neuroinflammatory diseases.

## Electronic supplementary material

Below is the link to the electronic supplementary material.

Supplementary Material 1: Figure S1. RHBDF2 knockdown reversed OGD/R treatment-induced inhibition of oxidative phosphorylation. a, Representative immunoblots of cytochrome c protein in cytoplasm or mitochondrion of HMC3 cells (Left). Quantitative analysis of cytochrome c expression in cytoplasm or mitochondrion of HMC3 cells (Right, N=3). b, ATP content in OGD/R-treated HMC3 cells (N=3). c, The level of ROS in OGD/R-treated HMC3 cells was determined by flow cytometry assay (Left). The mean fluorescence intensity (MFI) was used to reflect cellular ROS levels (Right, N=3). Data are presented as mean ± SD. In a, **p < 0.01 and ***p < 0.001 (OGD/R vs. Control). ##*p* < 0.01 (OGD/R+LV-shRHBDF2 vs. OGD/R+LV-shNC). In b and c, *p < 0.05, **p < 0.01, and ***p < 0.001.

Supplementary Material 2: Figure S2. Quantitative analysis of protein expression in animal experiment. a, Quantitative analysis of RHBDF2 protein expression of Figure 2c (N=6). b, Quantitative analysis of RHBDF2 protein expression of Figure 3c (N=6). c, Quantitative analysis of STING protein expression and p-IRF3/IRF3, p-TBK1/TBK1, and p-p65/p65 ratio of Figure 7a (N=6). Data are presented as mean ± SD. ns = not significant, *p < 0.05, and ***p < 0.001.

Supplementary Material 3: Figure S3. Quantitative analysis of protein expression in cell experiment. a, Quantitative analysis of RHBDF2 protein expression of Figure 8b (N=3). b, Quantitative analysis of iNOS protein expression of Figure 8d (N=3). c, Quantitative analysis of Arg1 protein expression of Figure 8g (N=3). d, Quantitative analysis of STING protein expression and p-IRF3/IRF3, p-TBK1/TBK1, and p-p65/p65 ratio of Figure 9c (N=3). e, Quantitative analysis of STING protein expression and p-TBK1/TBK1 ratio of Figure 10b (N=3). f, Quantitative analysis of iNOS protein expression of Figure 10c (N=3). g, Quantitative analysis of Arg1 protein expression of Figure 10d (N=3). Data are presented as mean ± SD. **p < 0.01 and ***p < 0.001.

Supplementary Material 4: Figure S4. Single-gene GSEA KEGG analysis of hub genes. a Single-gene GSEA KEGG analysis of IL-1B, VWF, COL1A1, COL1A2, IL7R, BMP2, and CSF3.

Supplementary Material 5: Figure S5. Single-gene GSEA GO analysis of hub genes. a Single-gene GSEA GO analysis of IL-1B, EGR2, VWF, COL1A1, COL1A2, and IL7R.

Supplementary Material 6

## Data Availability

No datasets were generated or analysed during the current study.

## Abbreviations

CIRI: Cerebral ischemia–reperfusion injury
RHBDF2: Rhomboid 5 homolog 2
MCAO/R: Middle cerebral artery occlusion/reperfusion
OGD/R: Oxygen-glucose deprivation/reoxygenation
NF-κB: Nuclear factor-κB
m6A: N6-methyladenosine
DEGs: Differentially expressed genes
IHC: Immunohistochemistry
IF: Immunofluorescence
qPCR: Quantitative real-time PCR
Me-RIP: Methylated RNA immunoprecipitation
