# Supplementary figures and images for: RHBDF2 governs microglial neuroinflammation during cerebral ischemia–reperfusion injury and is positively regulated by the m6A reader YTHDF1
Source: Mol Med. 2025 Sep 2;31:284. doi: 10.1186/s10020-025-01326-y (PMC12403488; doi:10.1186/s10020-025-01326-y)

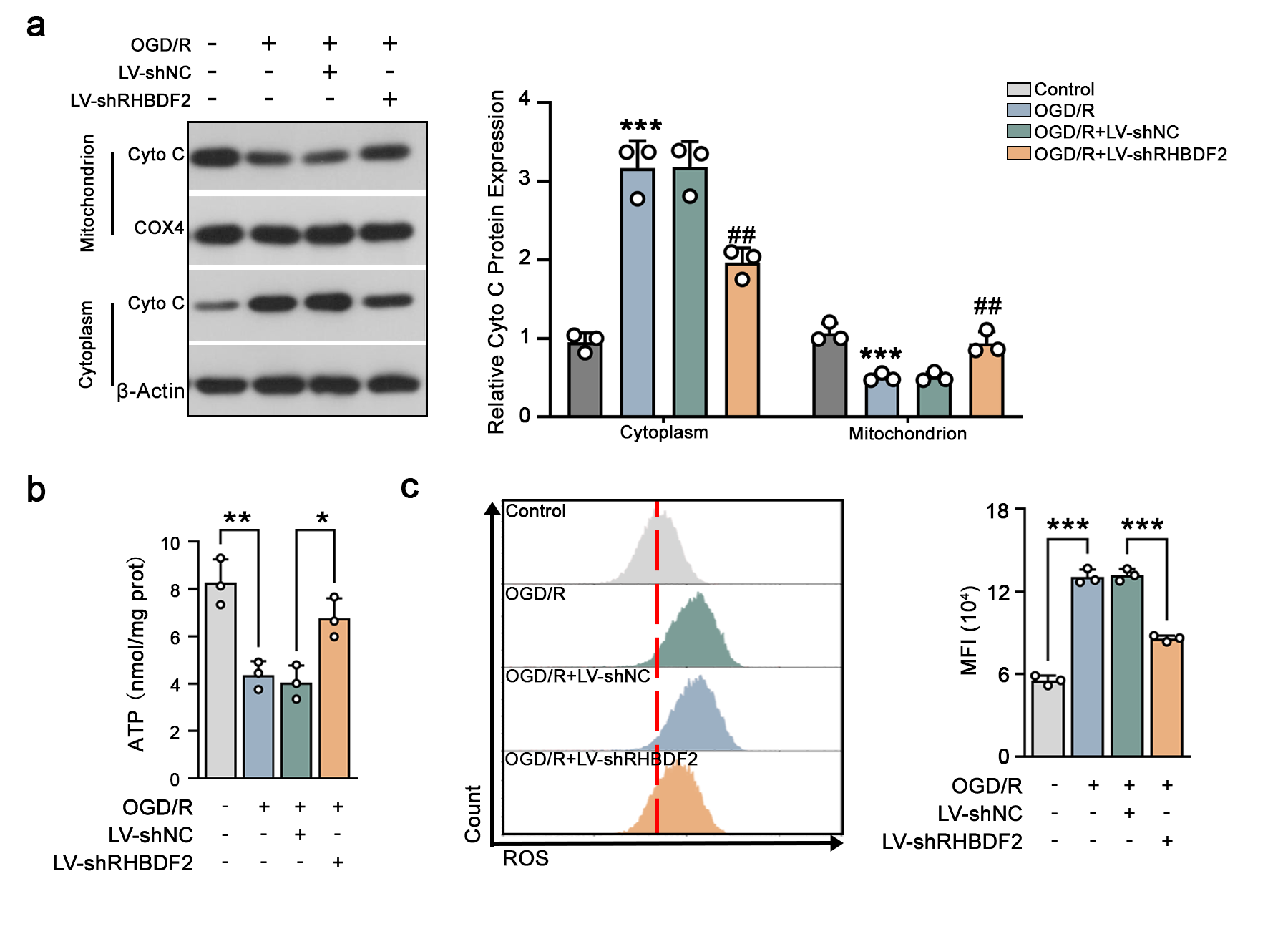

Supplement: Supplementary file 1 — Supplementary Material 1: Figure S1. RHBDF2 knockdown reversed OGD/R treatment-induced inhibition of oxidative phosphorylation. a, Representative immunoblots of cytochrome c protein in cytoplasm or mitochondrion of HMC3 cells (Left). Quantitative analysis of cytochrome c expression in cytoplasm or mitochondrion of HMC3 cells (Right, N=3). b, ATP content in OGD/R-treated HMC3 cells (N=3). c, The level of ROS in OGD/R-treated HMC3 cells was determined by flow cytometry assay (Left). The mean fluorescence intensity (MFI) was used to reflect cellular ROS levels (Right, N=3). Data are presented as mean ± SD. In a, **p < 0.01 and ***p < 0.001 (OGD/R vs. Control). ##p < 0.01 (OGD/R+LV-shRHBDF2 vs. OGD/R+LV-shNC). In b and c, *p < 0.05, **p < 0.01, and ***p < 0.001. [file 10020_2025_1326_MOESM1_ESM.tif]

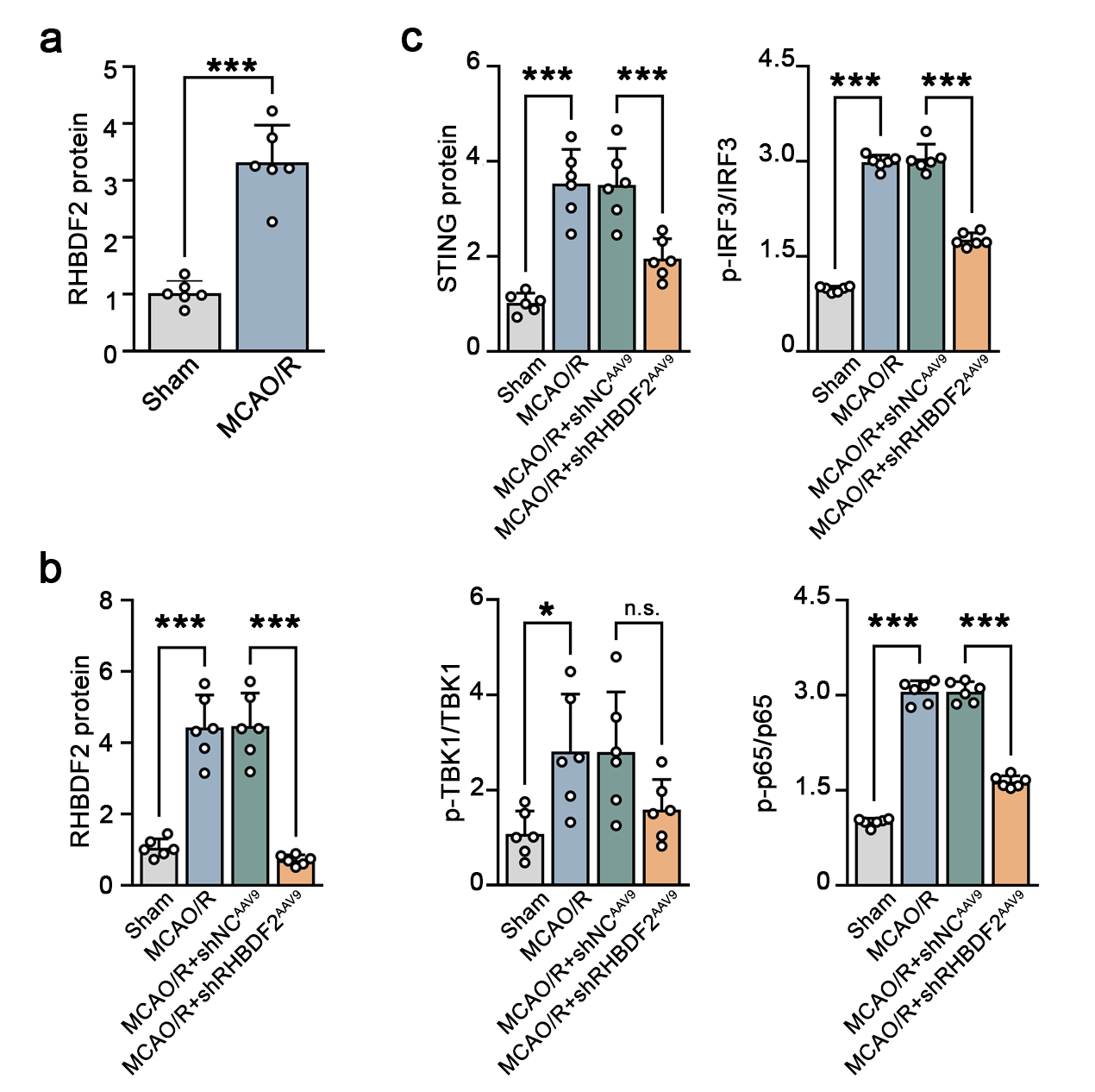

Supplement: Supplementary file 2 — Supplementary Material 2: Figure S2. Quantitative analysis of protein expression in animal experiment. a, Quantitative analysis of RHBDF2 protein expression of Figure 2c (N=6). b, Quantitative analysis of RHBDF2 protein expression of Figure 3c (N=6). c, Quantitative analysis of STING protein expression and p-IRF3/IRF3, p-TBK1/TBK1, and p-p65/p65 ratio of Figure 7a (N=6). Data are presented as mean ± SD. ns = not significant, *p < 0.05, and ***p < 0.001. [file 10020_2025_1326_MOESM2_ESM.tif]

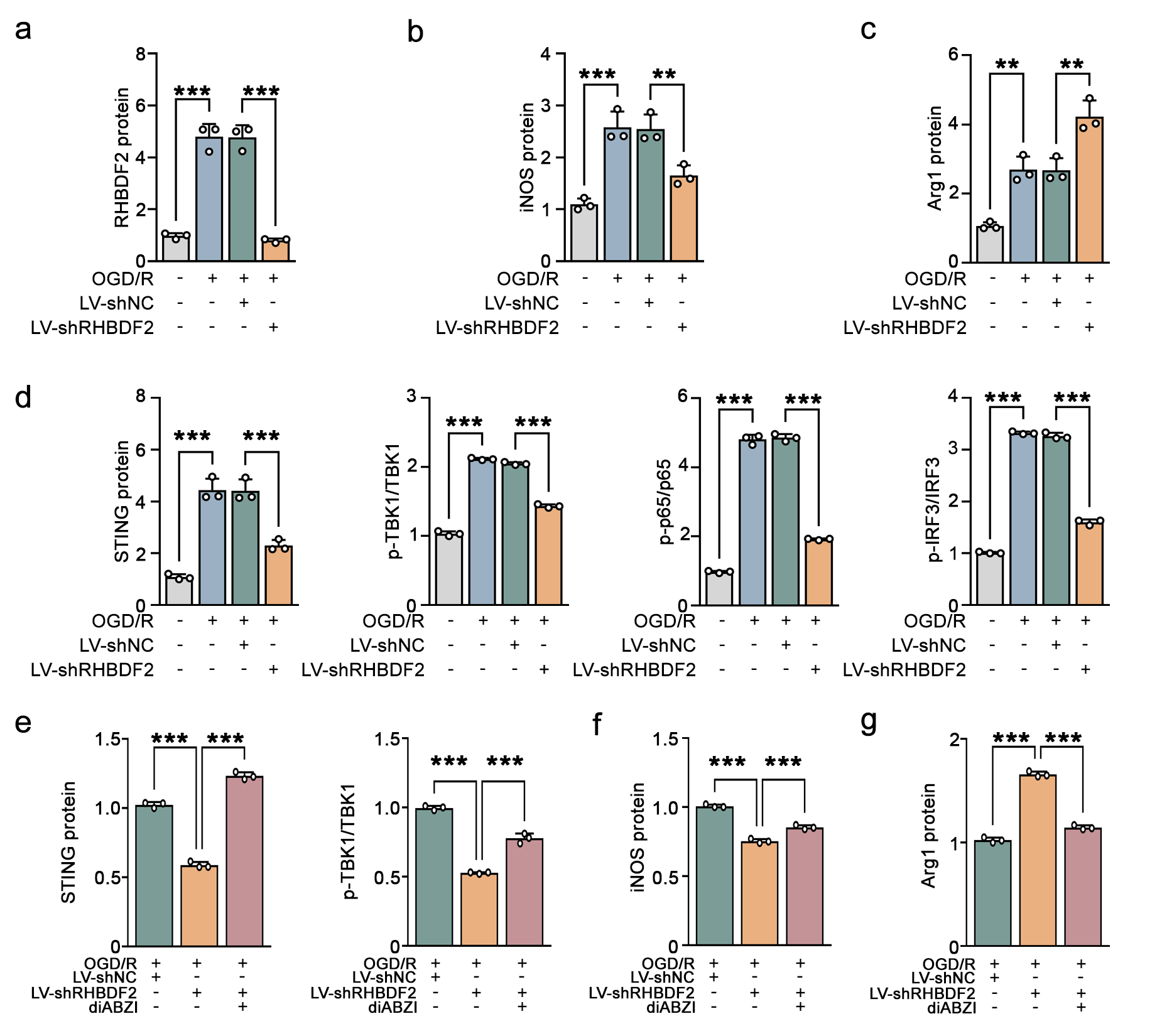

Supplement: Supplementary file 3 — Supplementary Material 3: Figure S3. Quantitative analysis of protein expression in cell experiment. a, Quantitative analysis of RHBDF2 protein expression of Figure 8b (N=3). b, Quantitative analysis of iNOS protein expression of Figure 8d (N=3). c, Quantitative analysis of Arg1 protein expression of Figure 8g (N=3). d, Quantitative analysis of STING protein expression and p-IRF3/IRF3, p-TBK1/TBK1, and p-p65/p65 ratio of Figure 9c (N=3). e, Quantitative analysis of STING protein expression and p-TBK1/TBK1 ratio of Figure 10b (N=3). f, Quantitative analysis of iNOS protein expression of Figure 10c (N=3). g, Quantitative analysis of Arg1 protein expression of Figure 10d (N=3). Data are presented as mean ± SD. **p < 0.01 and ***p < 0.001. [file 10020_2025_1326_MOESM3_ESM.tif]

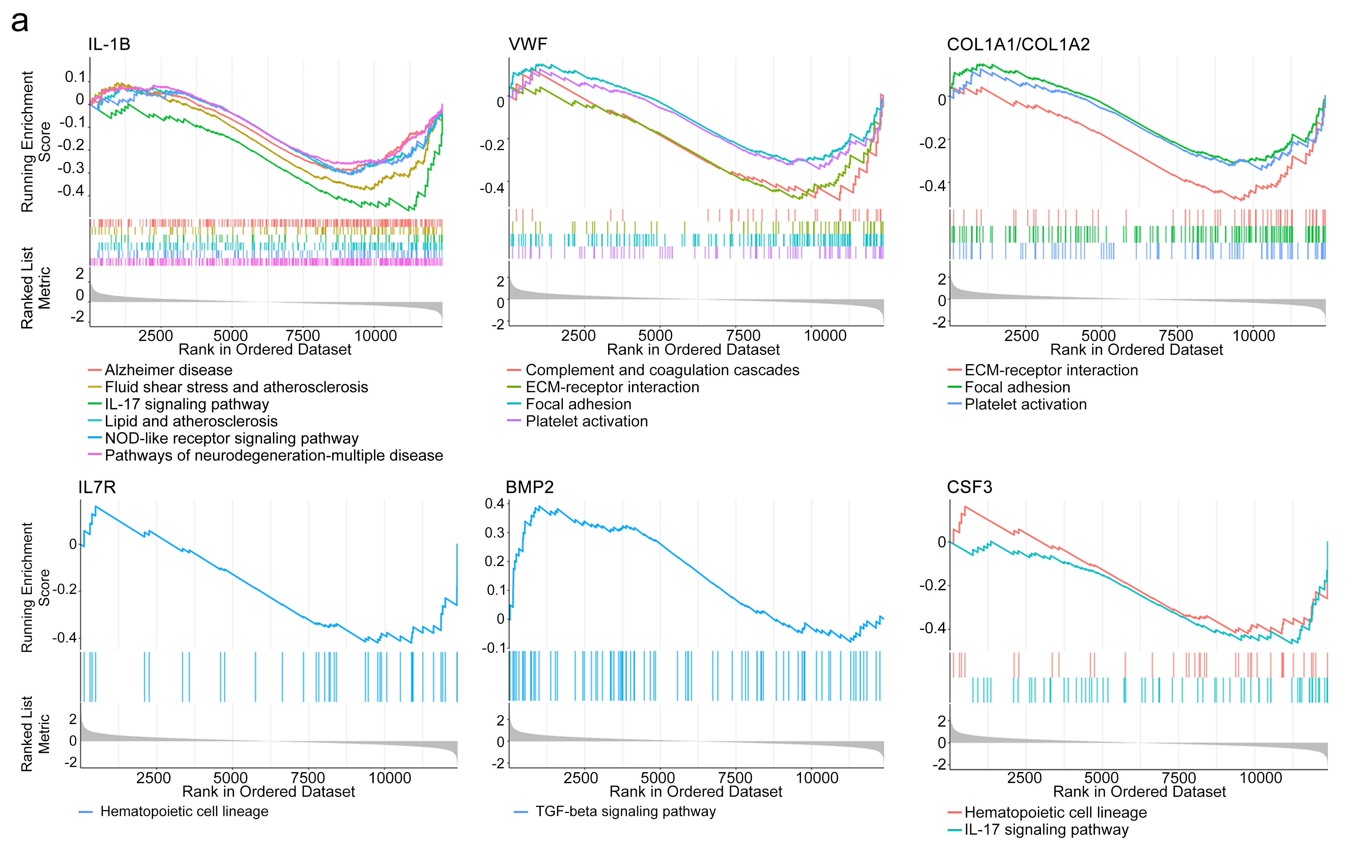

Supplement: Supplementary file 4 — Supplementary Material 4: Figure S4. Single-gene GSEA KEGG analysis of hub genes. a Single-gene GSEA KEGG analysis of IL-1B, VWF, COL1A1, COL1A2, IL7R, BMP2, and CSF3. [file 10020_2025_1326_MOESM4_ESM.tif]

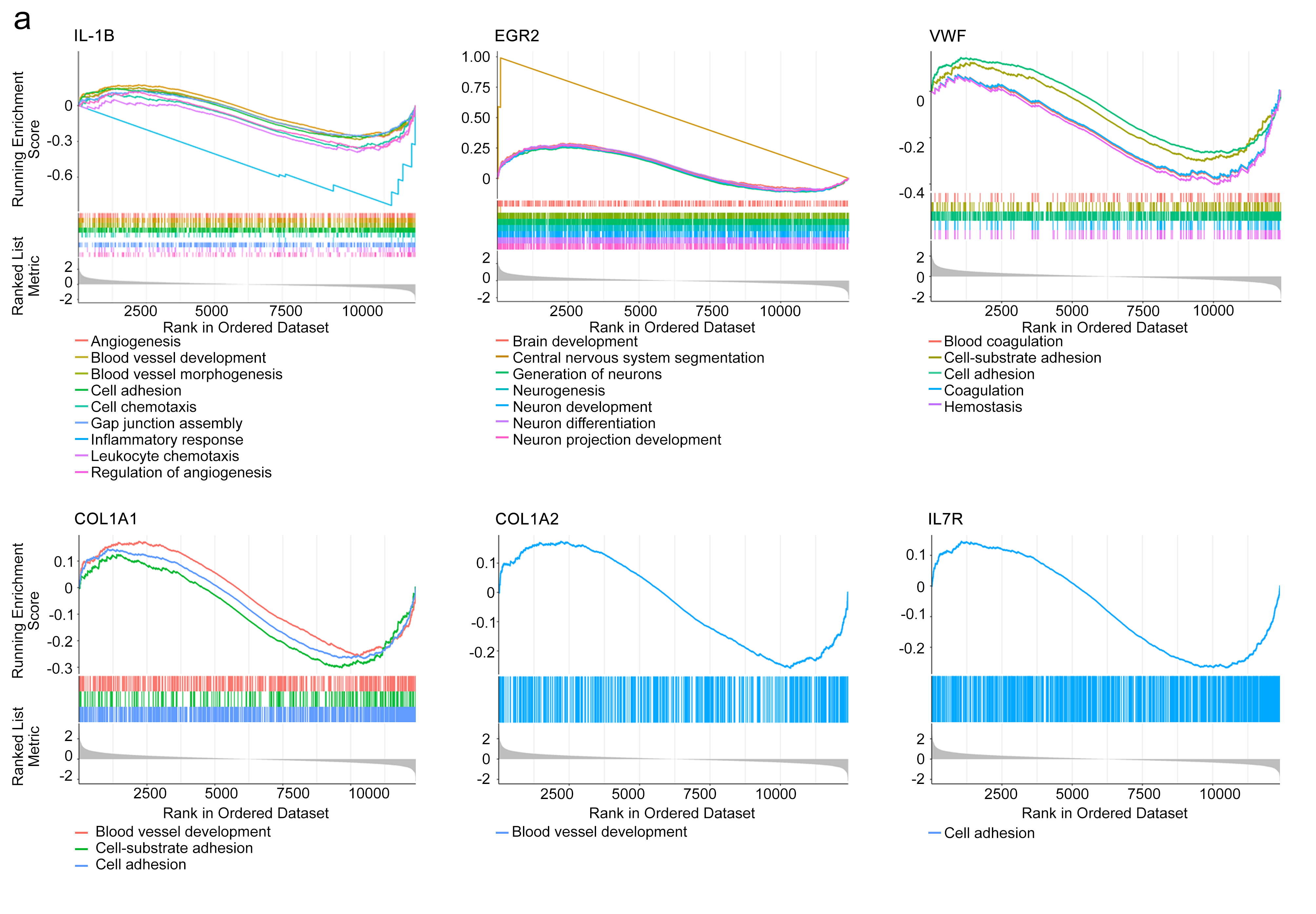

Supplement: Supplementary file 5 — Supplementary Material 5: Figure S5. Single-gene GSEA GO analysis of hub genes. a Single-gene GSEA GO analysis of IL-1B, EGR2, VWF, COL1A1, COL1A2, and IL7R. [file 10020_2025_1326_MOESM5_ESM.tif]

2c

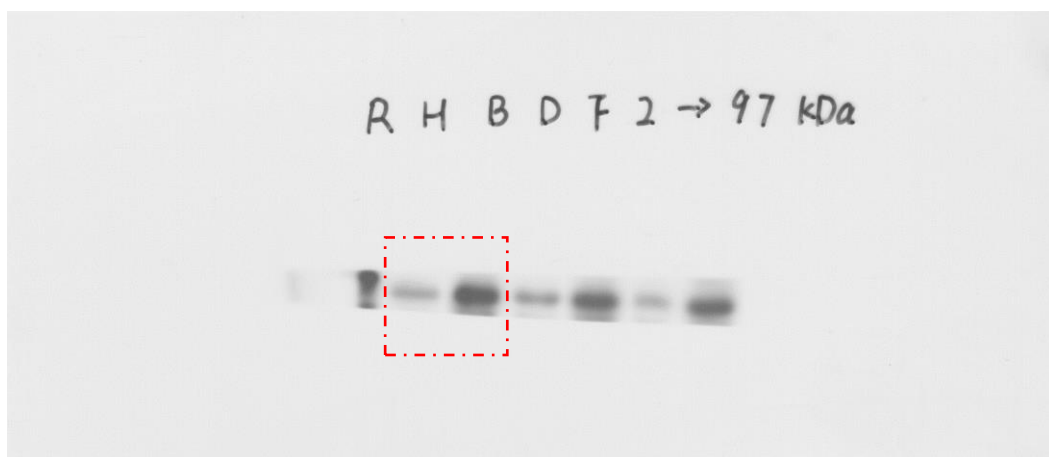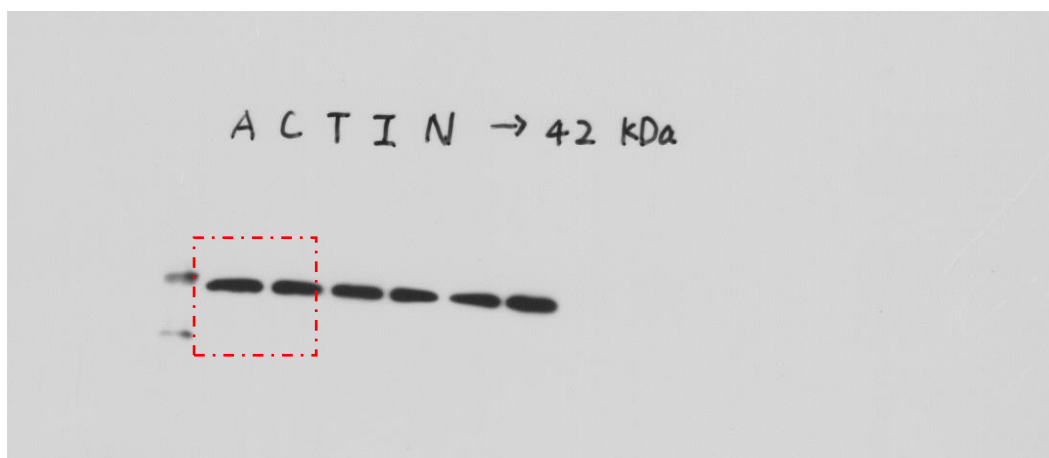

3c

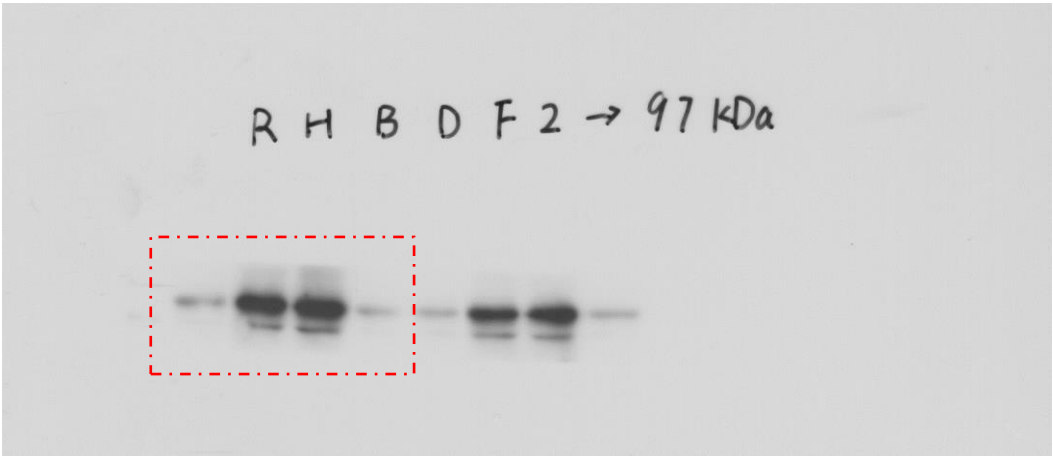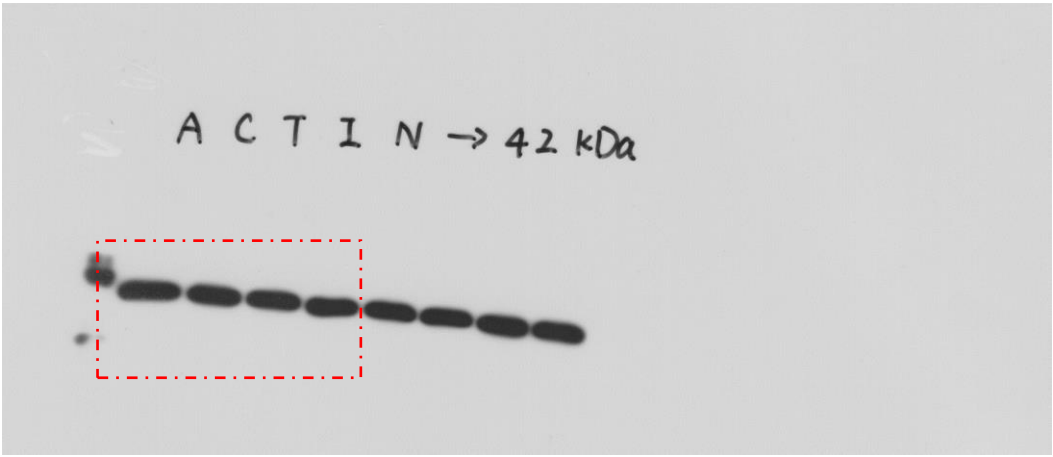

6c

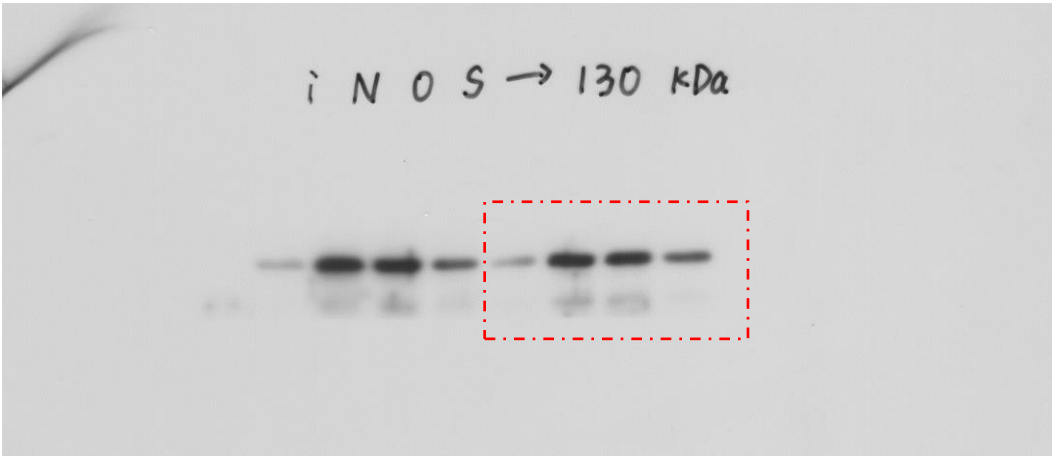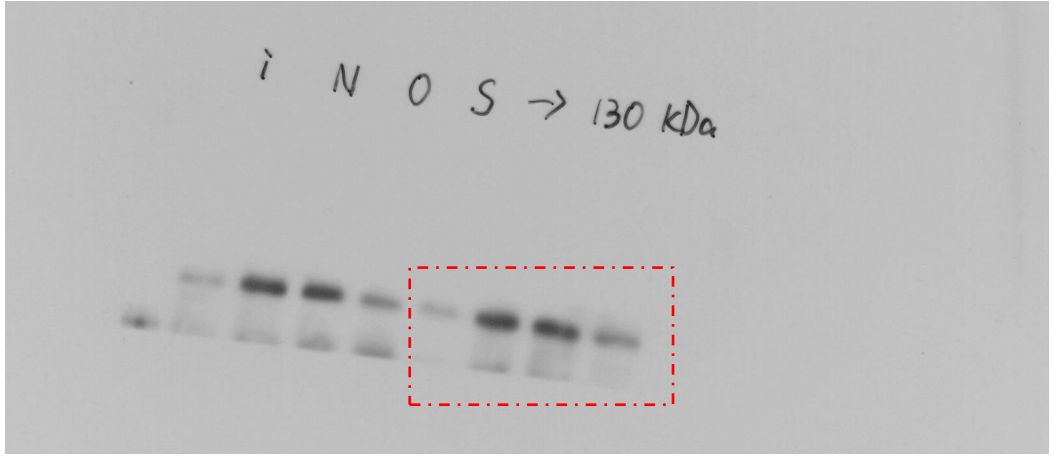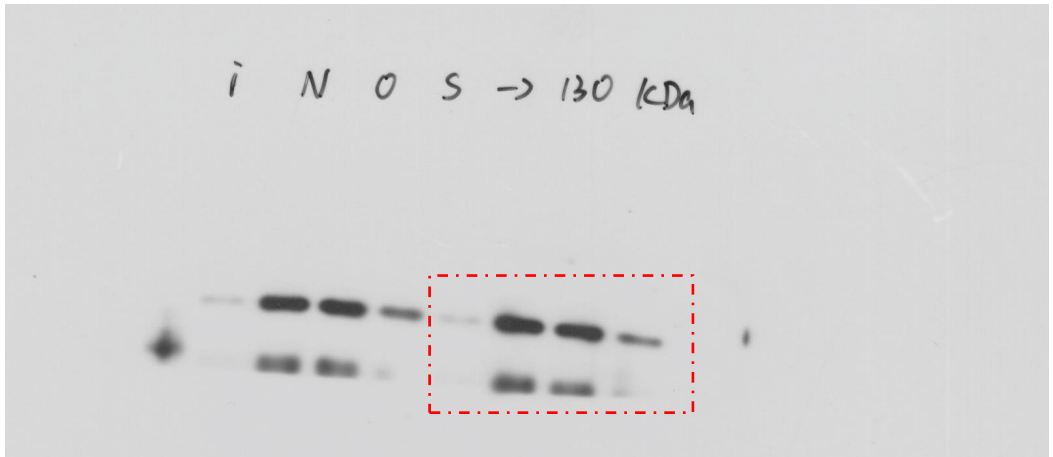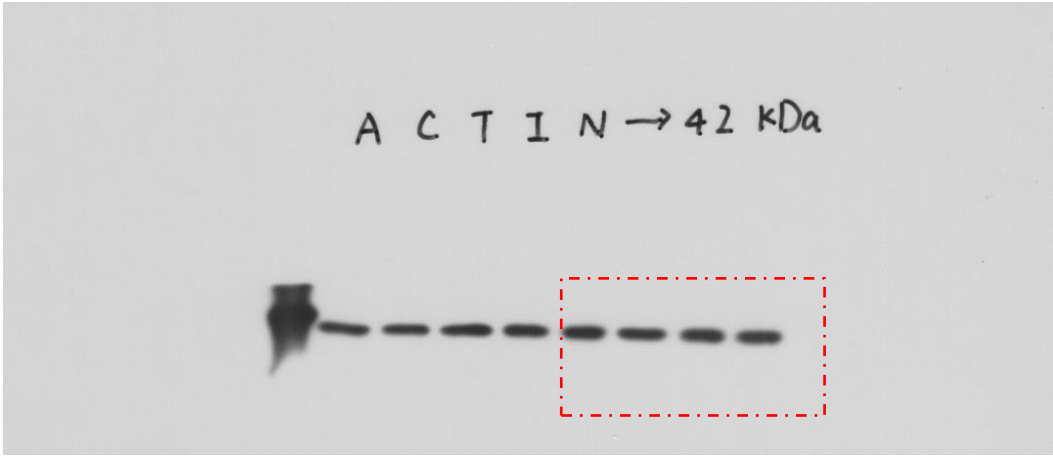

6e

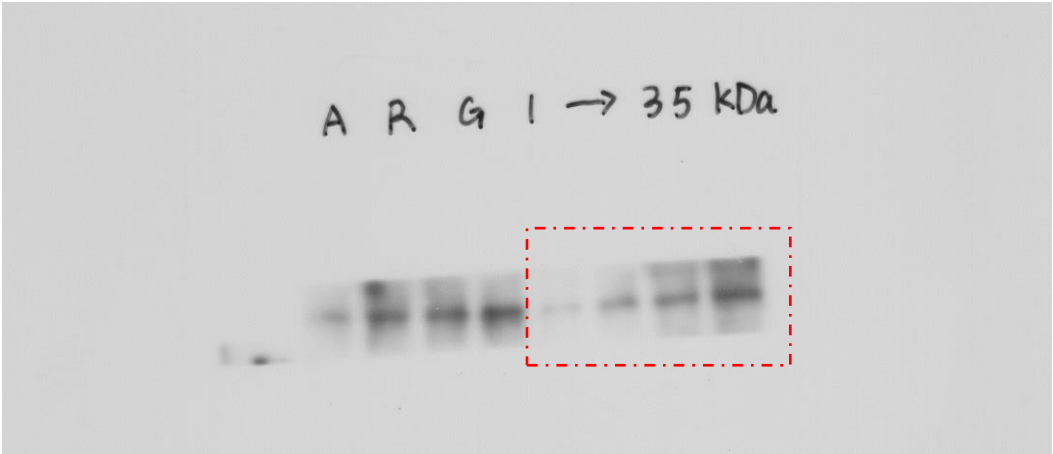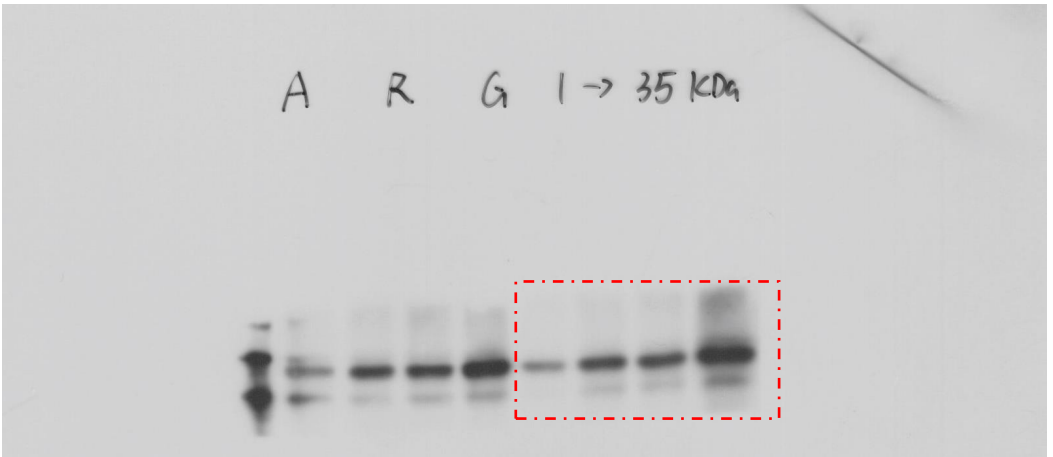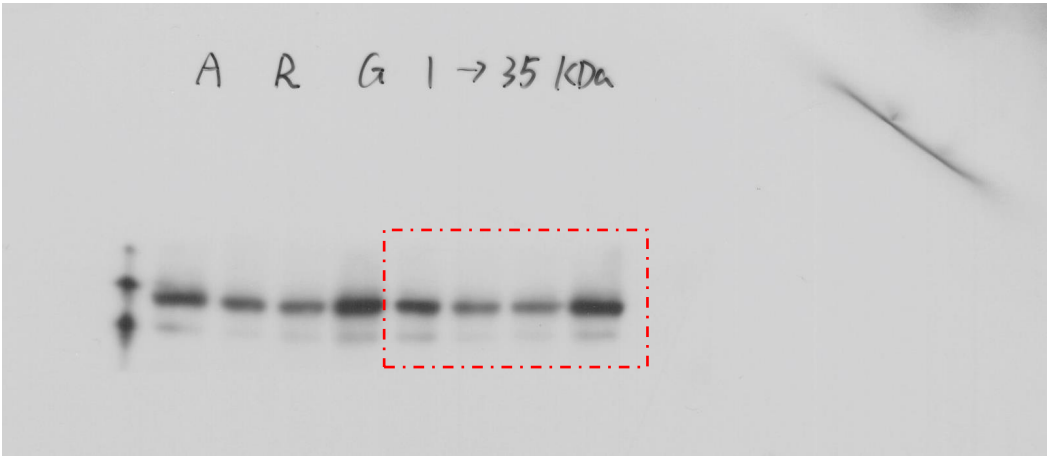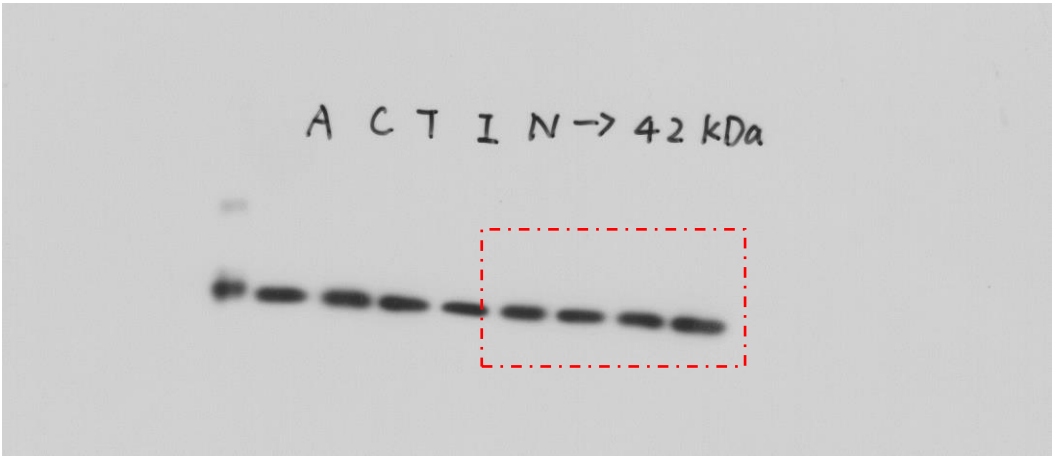

7a

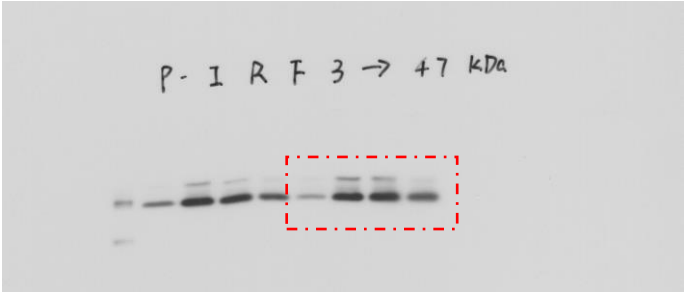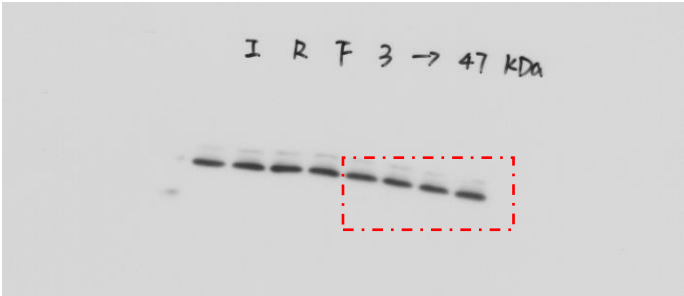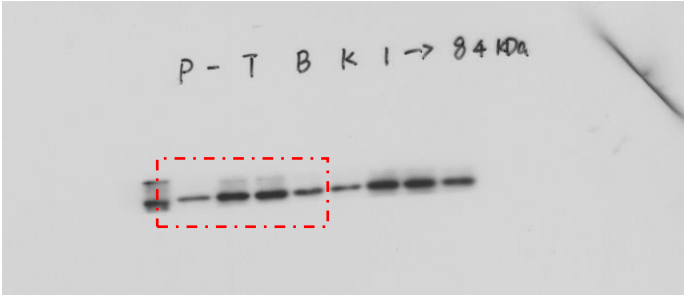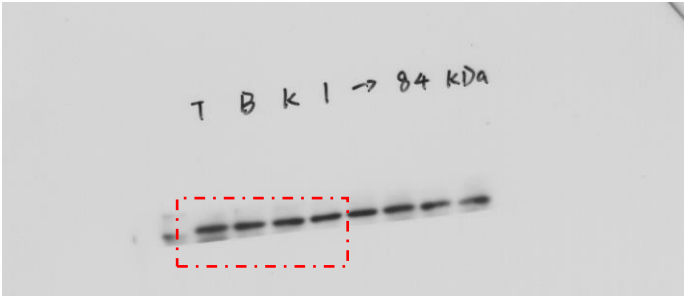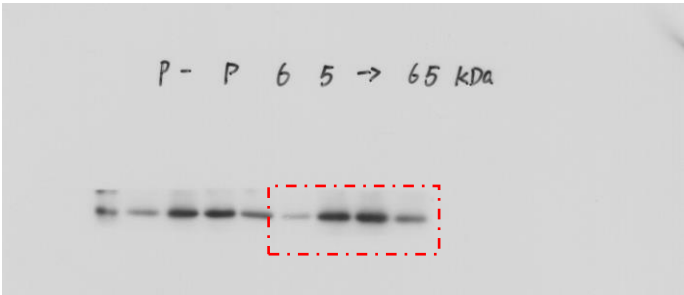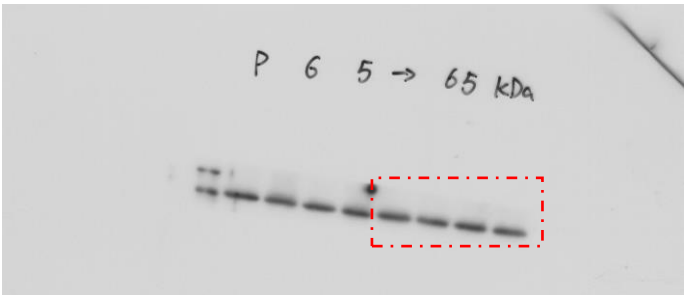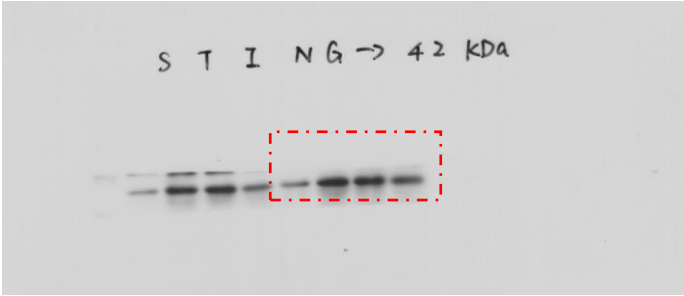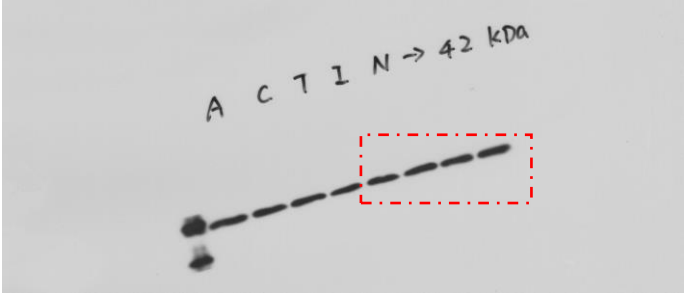

8b

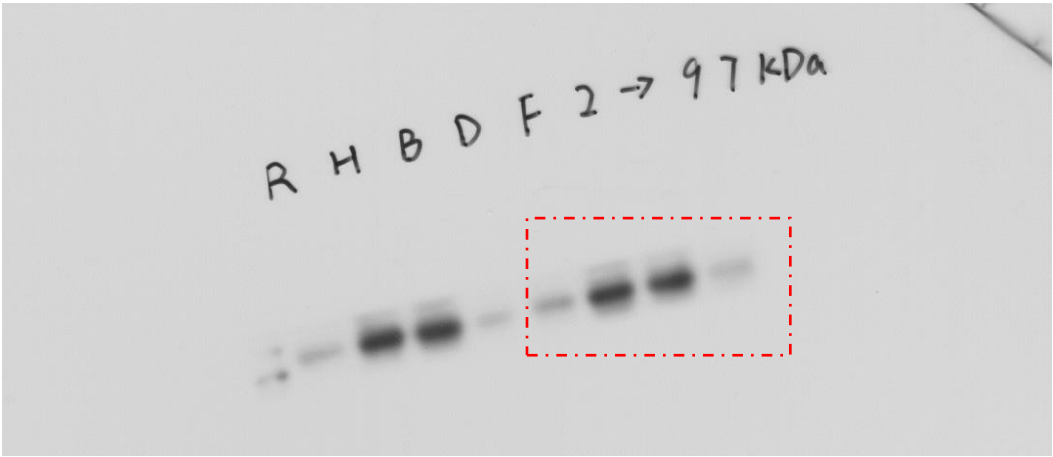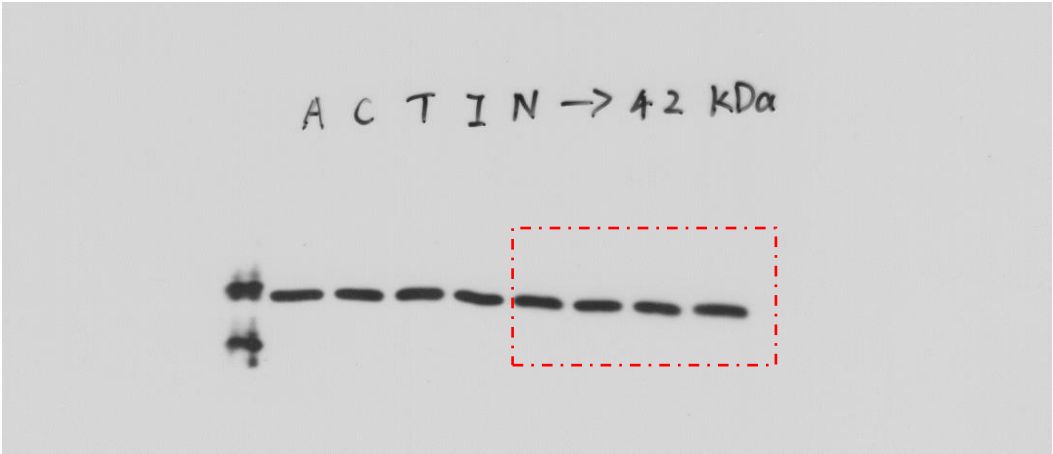

8d

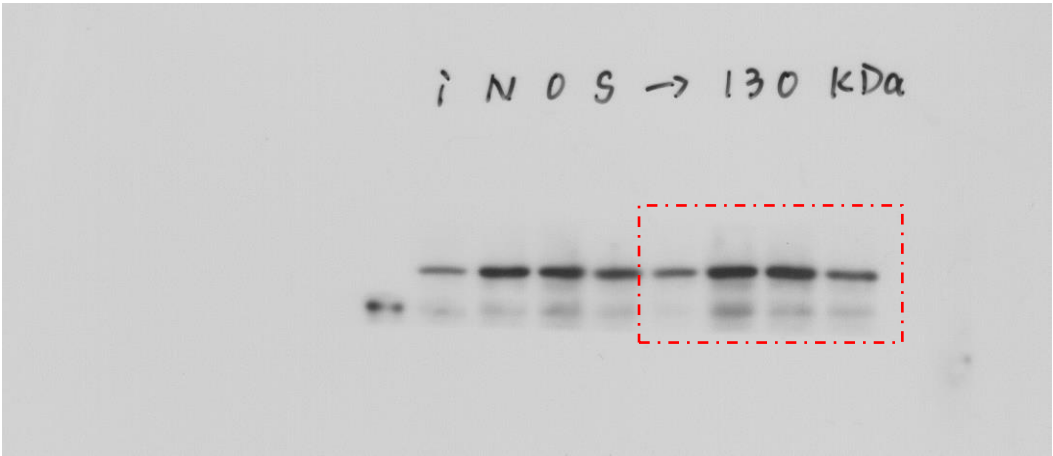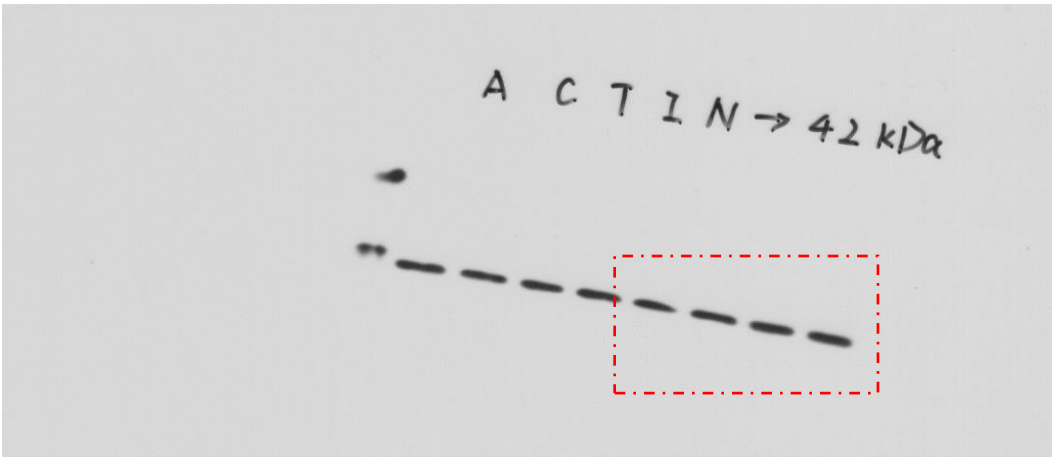

8g

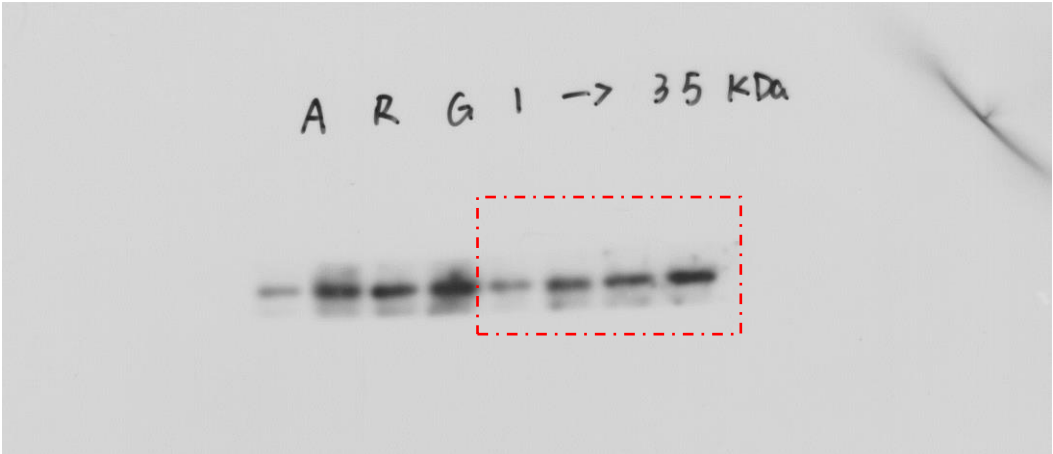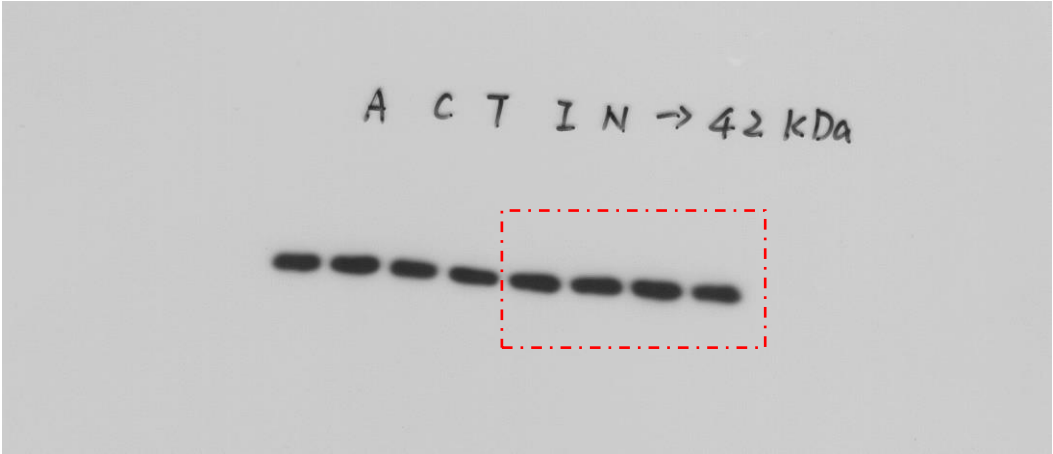

9a

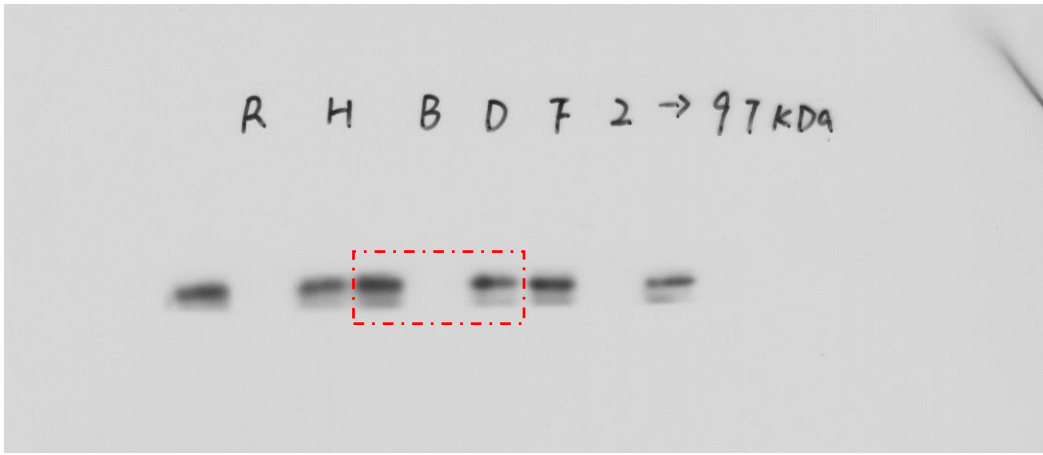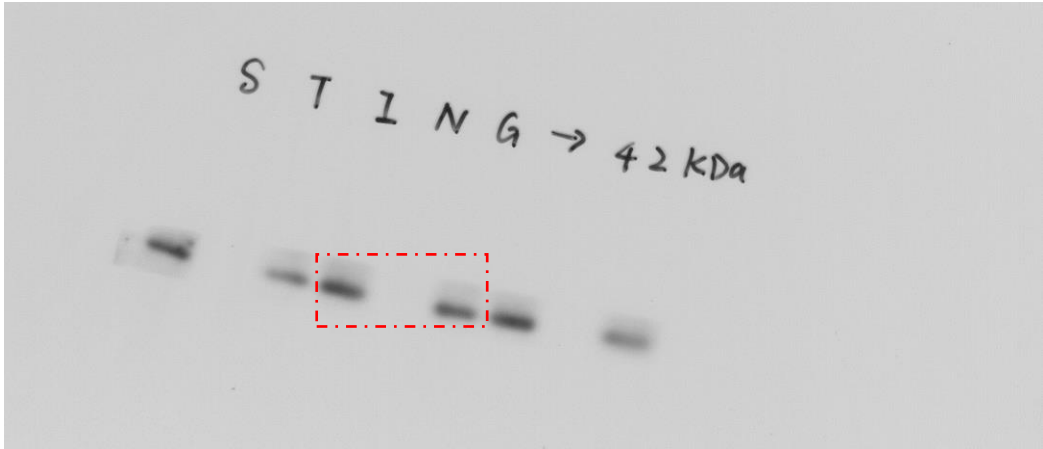

9c

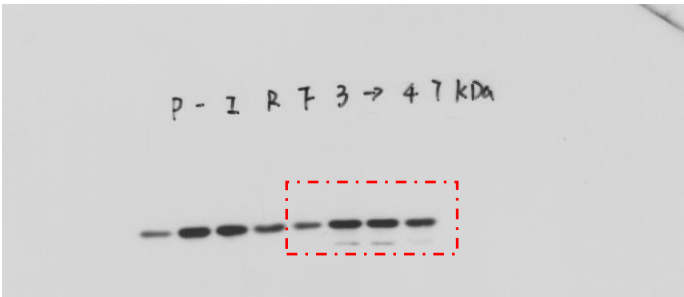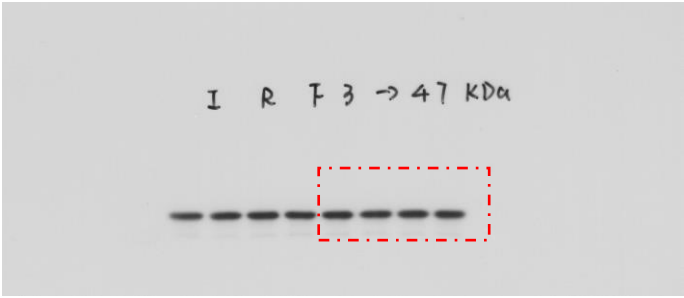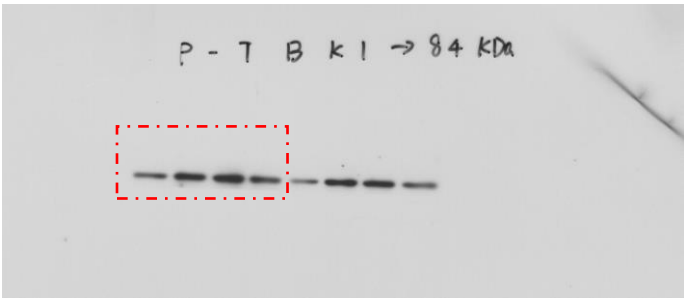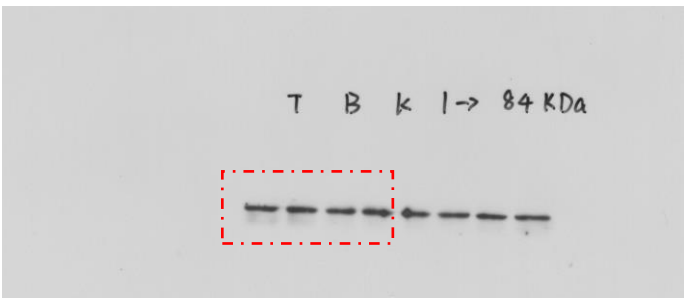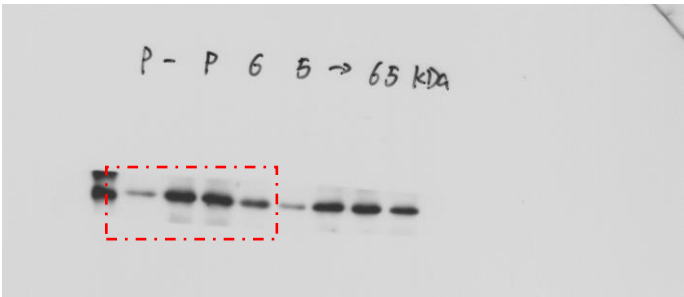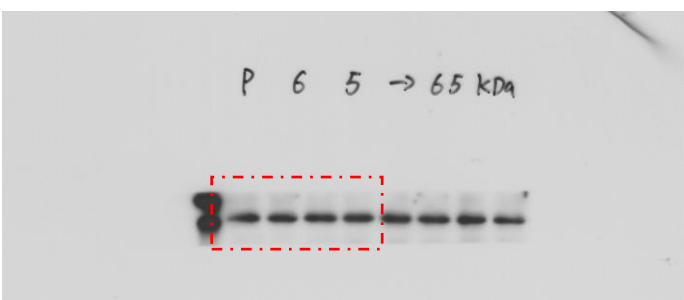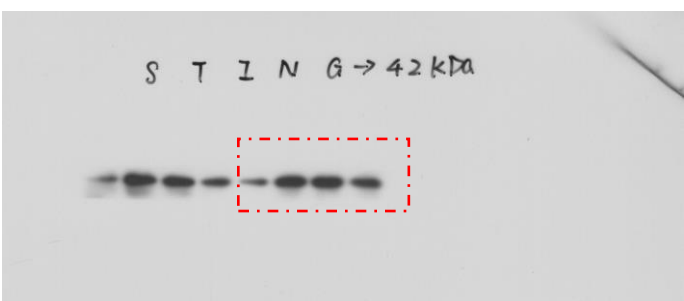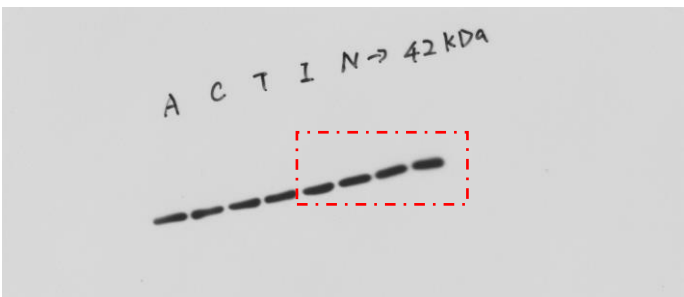

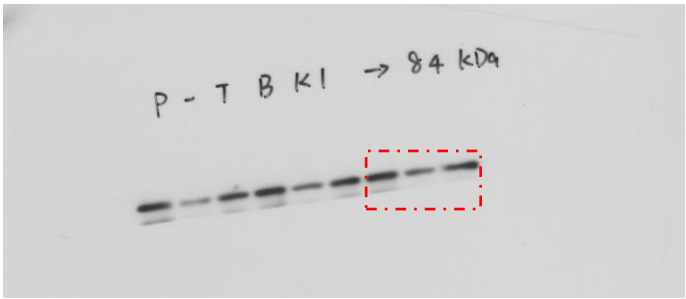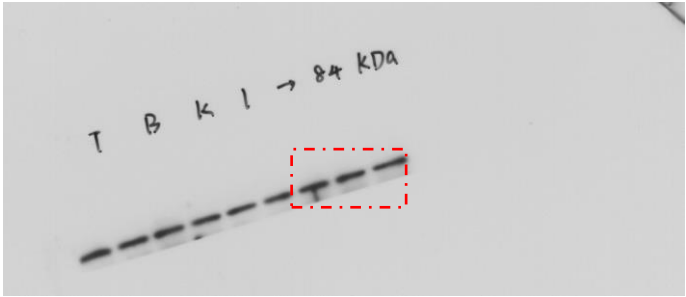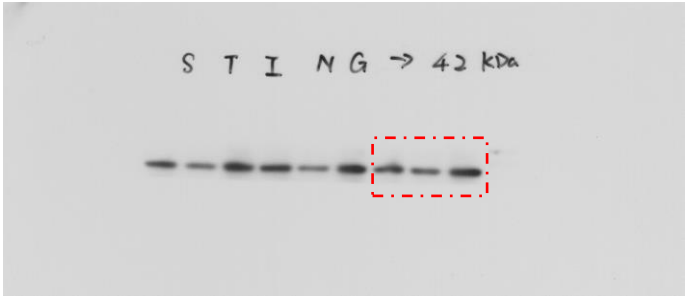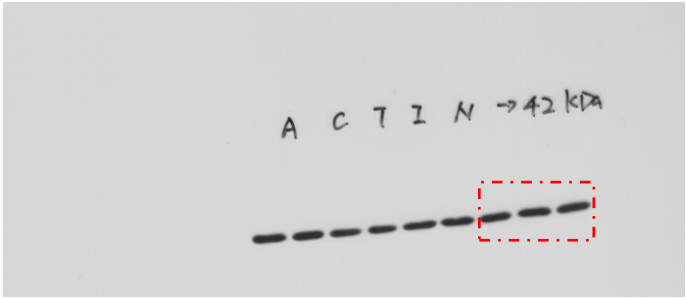

10c

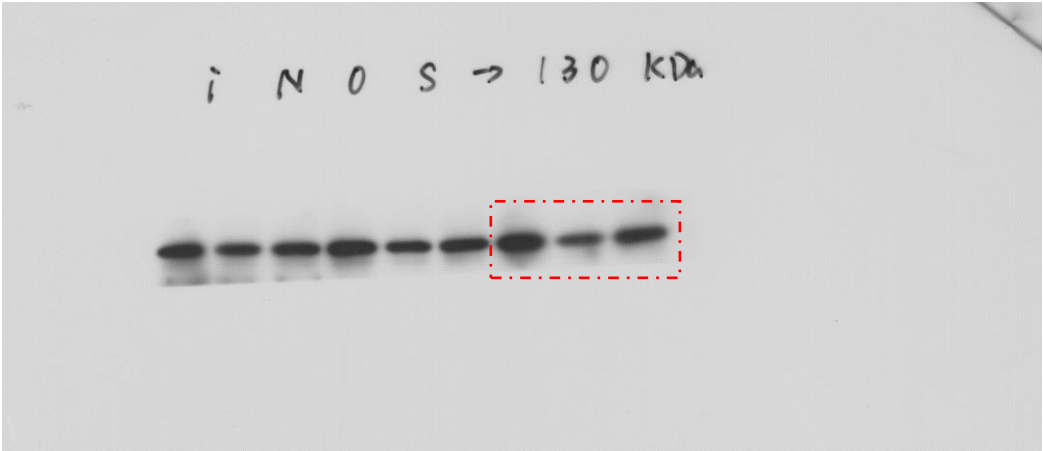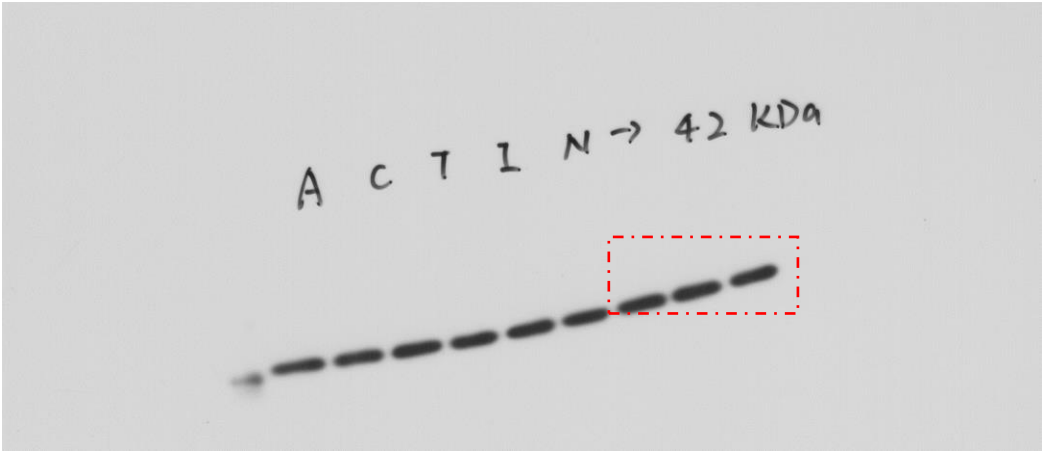

10d

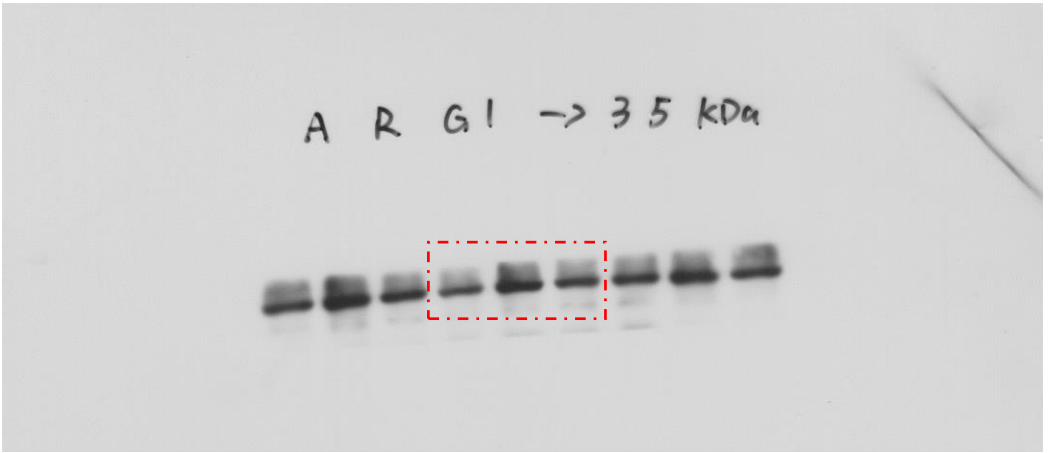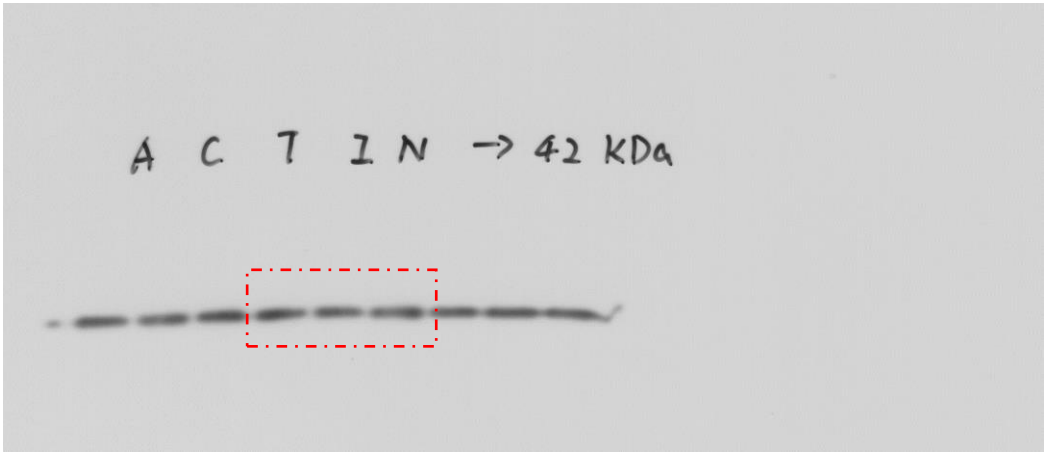

# 11f shYTHDF1

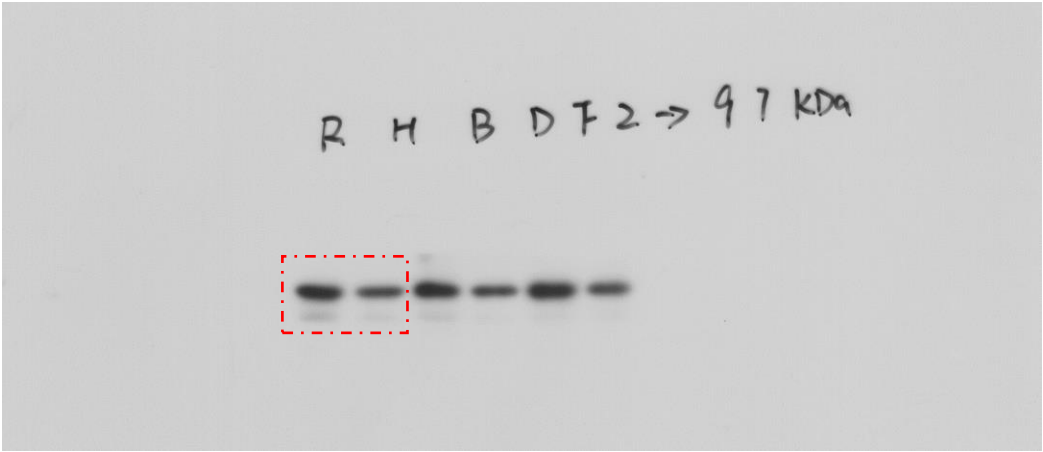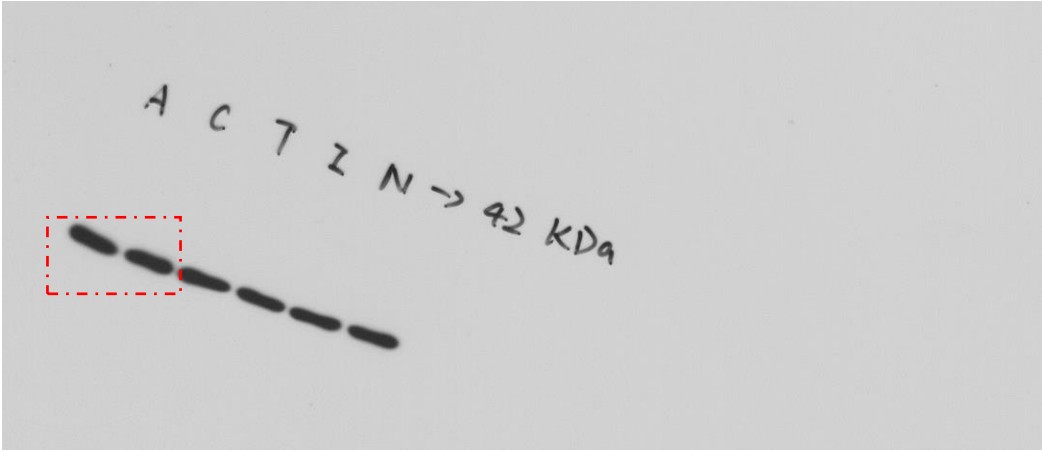

# 11f YTHDF1-OE

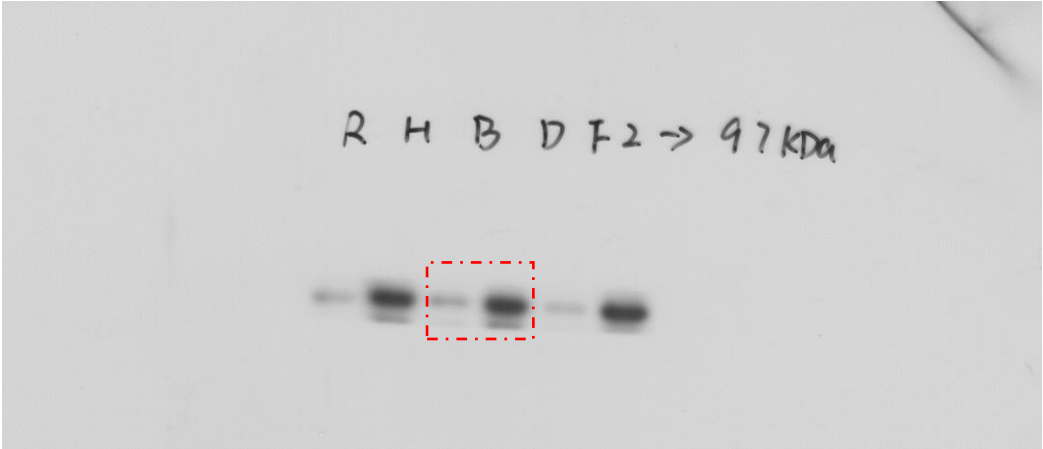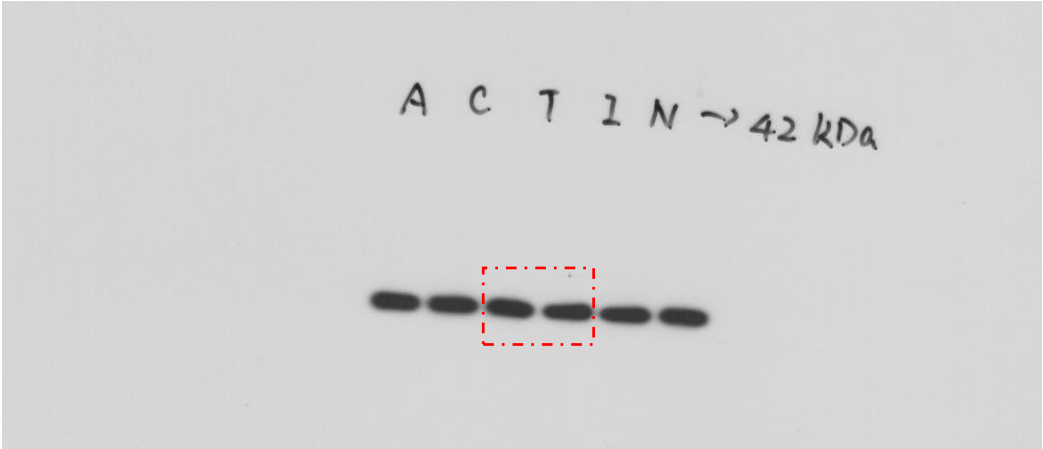

S1a

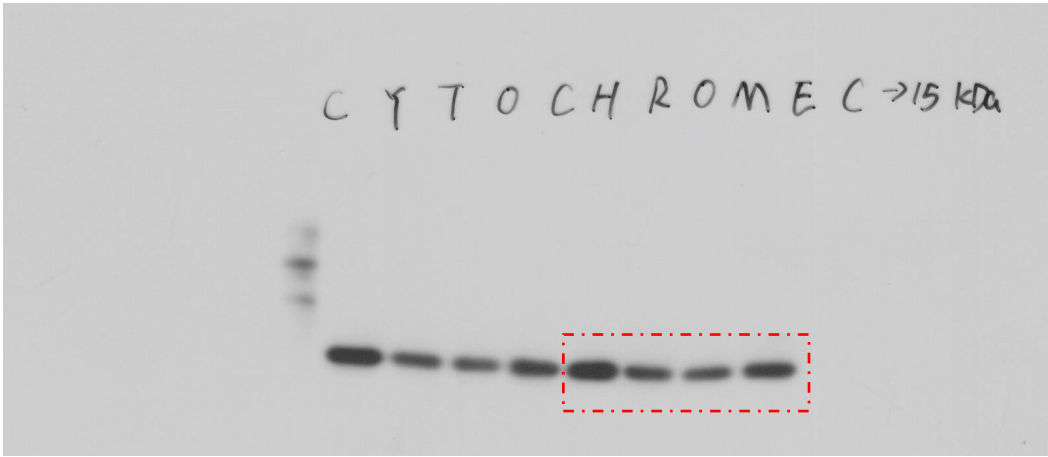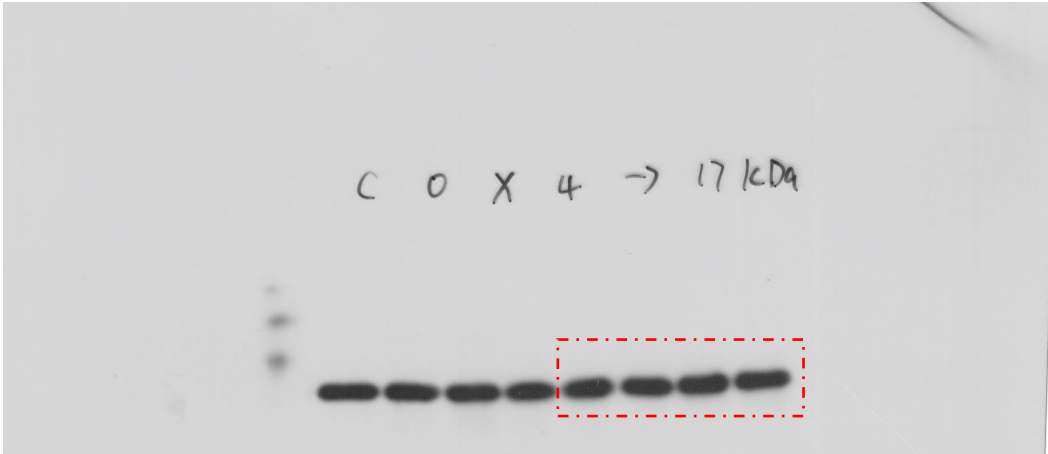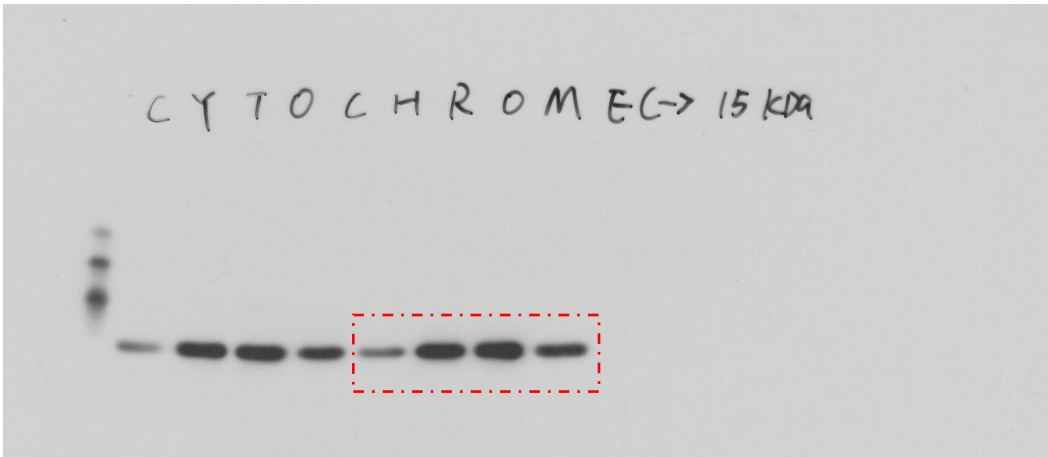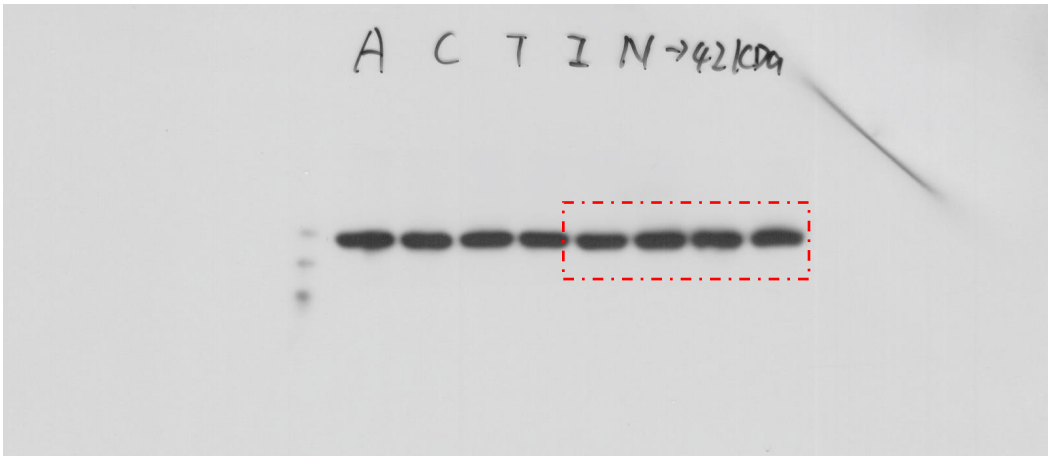

Supplement: Supplementary file 6 — Supplementary Material 6 [file 10020_2025_1326_MOESM6_ESM.pdf]
